# COVID-19 diagnosis within five days of symptoms onset among healthcare workers in Malawi; Non-randomized control trial of self-testing using Ag-RDTs

**DOI:** 10.1371/journal.pgph.0005604

**Published:** 2025-12-16

**Authors:** Chimwemwe Kwanjo Banda, Wezzie S. Lora, Lucky G. Ngwira, Yasmin Dunkley, Emily Nightingale, Hendricks Mwenelupembe, Richard Chilongosi, Gabrielle Bonnet, Nicola Desmond, Elizabeth L. Corbett, Karin Hatzhold, Augustine T. Choko

**Affiliations:** 1 Department of Public Health, Malawi Liverpool Wellcome Trust Clinical Research Programme (MLW), Blantyre, Malawi; 2 Department of Economics and Policy, Kamuzu University of Health Sciences (KUHeS), Blantyre, Malawi; 3 Department of Clinical Research, London School of Hygiene and Tropical Medicine, London, United Kingdom; 4 Family Health Services, Blantyre, Malawi; 5 Department of Infectious Disease Epidemiology, London School of Hygiene and Tropical Medicine, London, United Kingdom; 6 Department of International Public Health, Liverpool School of Tropical Medicine, Liverpool, United Kingdom; 7 Population Services International Global, Washington, DC, United States of America; Ex DNDi, UNITED STATES OF AMERICA

## Abstract

Efficient and sustainable models of Corona Virus Disease of 2019 (COVID‑19) screening among healthcare workers (HCWs) such as self-testing are essential for averting transmission within and outside health care facilities. We compared the number of confirmed early COVID‑19 positive diagnoses (defined as COVID‑19 cases diagnosed within five days of symptoms onset) among HCW in self-testing arm using Antigen Rapid Diagnostic Test kits (Ag-RDT) and standard of care arm (SOC) who were offered COVID‑19 testing if they presented at the clinic with any COVID‑19 symptoms. Twelve primary healthcare facilities in Blantyre, Malawi, were purposively allocated (1:1) aiming for geographical and size balance in a 2-arm non-randomised cluster trial (ISRCTN: 17596113), available from https://doi.org/10.1186/ISRCTN17596113. Arm-1 was SOC and Arm-2 was COVID 19 self-testing (C19ST). HCWs in the C19ST arm had twice-weekly COVID‑19 Ag-RDT self-testing. The primary outcome compared by arm the harmonic mean number of early COVID‑19 positive diagnoses among HCWs. Analysis was by intention-to-treat using cluster-level summaries and t-test, with adjustment for imbalance. Participation was 99.8% among eligible HCWs across all facilities (1081/1083). Of the 1081 participating, 612 (56.6%) and 469 (43.4%) were in SOC and C19ST arm, respectively. Mean age was 35.5y (sd: 9.3); 183/612 (29.9%) in SOC were male, compared to 166/469 (35.3%) in C19ST; overall prior vaccination was 80.0% with no difference between SOC, (81.5%) and C19ST (78.0%). Follow-up at exit (12 weeks) was high (SOC: [94%]; C19ST: [87.6%]) and a harmonic mean of 1 and 4 HCWs had early COVID‑19 diagnosis in SOC and C19ST arms, respectively. COVID‑19 self-testing using Ag-RDTs provided a safe, quick, and reliable model for identifying early-onset symptomatic and asymptomatic COVID‑19 positive HCW. Self-testing was feasible to integrate for routine screening among HCW potentially reducing disruption to health services. This model has potential for wide scale up programmatically especially in resource-constrained settings.

## Introduction

Now that the Corona Virus Disease of 2019 (COVID‑19) pandemic transitioned into an endemic situation, the significance of and investment in testing for infection control diminished [1]. In many low and middle income countries (LMICs) such as Malawi, testing capacity never reached a sufficient threshold for population level infection control, with delivery models focused on provider delivered diagnostics (polymerase chain reaction [PCR] initially then Antigen Rapid Diagnostic Test [Ag-RDT]). Self-testing could however have played a pivotal role in the pandemic (and did so in Europe and North America) with the following benefits: early diagnosis; increased diagnostic capacity for COVID‑19; reduced access barriers; and was particularly useful in screening of healthcare workers (HCW) for early detection of COVID‑19 infection prior to symptom onset, which enables early isolation and prevention of spread of the virus to patients and other HCW [2,3]. Even in the post-pandemic period, evaluating the use of self-testing in HCW provides valuable evidence on feasibility, acceptability, and adherence to testing strategies that operate outside of traditional provider delivered models, and may guide in future pandemic preparedness [1].

The optimisation of delivery strategies for vulnerable people in LMICs for self-testing was also part of a call by the World Health Organization (WHO) for evidence generation [4]. Studies in Europe have demonstrated a high diagnostic accuracy of Ag-RDT for COVID‑19 among HCW regardless of whether they were clinically trained or not [2,3]. COVID‑19 self-testing has so far been widely implemented and made available in high income countries, and its performance (sensitivity and specificity), and usability has already been ascertained in Malawi [5]. That self-testing did not play such a key role in LMICs speaks to the ever-changing nature of the COVID‑19 pandemic and lack of policy guidelines and recommendations on optimal approaches to deliver COVID‑19 Ag-RDT self-test kits [6].

This research aims to investigate potentially sustainable distribution models for delivering COVID‑19 self-testing to inform evidence-based approaches for future pandemic preparedness. Specifically, we compare the number of confirmed COVID‑19 cases diagnosed within five days of symptoms onset, and the number of days off due to suspected or confirmed COVID‑19 between the COVID‑19 self-testing (C19ST) and standard of care (SOC) study arms in Blantyre, Malawi. This work was part of a larger consortium project implemented in Africa, Asia and America for COVID‑19 preparedness (abbreviated as 3ACP) aimed at generating evidence on which to base self-testing recommendations in view of existing knowledge gaps in this field.

## Methods

### Ethics statement

Ethics approval for the study was obtained from Kamuzu University of Health Science’s College of Medicine Research and Ethics Committee (COMREC) in Malawi (P.05/22/3649), and internationally from the World Health Organization Ethics Review Committee in Geneva, Switzerland (CERC.0163), and the London School of Hygiene & Tropical Medicine Ethics Committee (26874). Permission to access the 12 participating health facilities was obtained from the director of health and social services for Blantyre.

### Study design and population

We conducted a 2-arm non-randomised cluster trial among HCW to compare the number of confirmed COVID‑19 cases diagnosed within 5 days of symptoms onset, and the number of days off due to suspected or confirmed COVID‑19 between the C19ST and SOC study arms (clinical trial registration number: SRCTN17596113). Recruitments took place between 11^th^ January, 2023 and 31^st^ May, 2023 during week days (Monday to Friday), among HCW working at 12 public primary care facilities recommended by the Blantyre District Health Office (DHO) based on the local needs. At the time the study was conducted, COVID‑19 testing of any kind was not available at primary care facilities in the country. During the study period, occupational diagnostic COVID‑19 testing with COVID‑19 Ag-RDTs that was introduced to all 12 facilities and was made available to staff and clients seeking Outpatient Department (OPD) care requiring the test. In addition to the COVID‑19 Ag-RDTs available at the facility, HCW at six self-testing health facilities (CT19ST arm), were offered twice-weekly self-testing for early diagnosis and infection prevention control. Before the names of participating facilities were known, a standard operating procedure (SOP) for facility allocation was developed to guide a random and unbiased assignment process. Following this SOP, each facility was first paired with a similar facility based on location (commercial area, urban, peri-urban, or rural) and facility size. The pairs were then arranged in alphabetical order. Within each pair, the facility that appeared first alphabetically was assigned to the CT19ST arm, while the second facility was assigned to the SOC arm.

Upon receiving approvals from all relevant authorities, we conducted meetings with staff at each of the facilities to introduce the study, the intervention, directed interested participants on where they could meet with study staff. HCW interested to participate approached field research assistants in a private room at (OPD) where they were given more information about the study and screened for eligibility. HCW were eligible if: a) they were aged 18 years and above; b) working at the facility and included nurses, doctors, clinic assistants, health surveillance assistants, clinical officers, and support staff such as data clerks and porters; and c) known to be available at the facility for the next 12 weeks. Written or thumb print informed consents were obtained from all eligible participants who accepted to take participate in the study. Participants recruited in the C19ST arm were given a participation card for recording of collection of self-test kits (S1 Text). With an assumed harmonic mean number of 20 and 35 HCWs (common standard deviation: 12) in SOC and the C19ST arm confirmed (either by PCR or by repeat professional Ag-RDT) COVID‑19 diagnosed within three months, 6 clusters per arm of ~100 HCWs each, the study would have 84.1% power to detect the stated difference of harmonic count of 15. A total of 1081 HCW (C19ST arm n = 469; SOC arm n = 612) were recruited from the 12 facilities.

### Intervention

We partnered with the Ministry of Health (MoH) and Population Services International (PSI) to provide the intervention. In the six facilities allocated to the C19ST arm, each participating HCWs collected two COVID‑19 Ag-RDT self-test kits per week for 12 weeks from trained MoH distributors at a designated point within the facility (either at the OPD area or laboratory). The distributors were MoH employed staff from the same facility and included support staff such as lab assistants, health surveillance assistants, patient attendants, hospital attendants and social workers. PSI, was responsible for all implementation components, including training and provision of kits and masks. ACON Flow*flex*™ COVID‑19 Antigen Home Test for self-testing were used for the intervention [7]. According to the manufactures, the test kits have a sensitivity of 97.1% and specificity of 99,6% [8]. Before the test kits were used, an accuracy assessment was carried out by National Health Reference Laboratory (NHRL) of Public Health Institute of Malawi (PHIM) as the test was not under WHO prequalified list of test kits, which it passed. All HCWs who self-tested positive were asked to confirm infection with anterior nasal swab for repeat professional use Ag-RDT and received high quality masks for their contacts as well as themselves.

### Exposure and outcome measurement

We adopted questions from the COVID‑19 questions bank [9] to develop three data tools to collect; a) baseline data at recruitment; b) follow-up data at 4 and 8 weeks; c) collect exit data at 12 weeks. The baseline data tool solicited information about participants’ sociodemographic characteristics; assessed the participant for their risk for severe COVID‑19 (including vaccine status, comorbidities, and body mass index [BMI]); participants’ knowledge on basic facts about COVID‑19; and participant’s practices and behaviors regarding COVID‑19 prevention. The follow-up data tools assessed if participants suffered from any illness in the past 4 weeks. If they did, further questions were asked to assess: COVID‑19 symptoms in the previous 4 weeks; management and care if there were any reported symptoms; and case classification of the participant if they are a confirmed case of COVID‑19 and if asymptomatic. The exit data tool re-assessed participants’ COVID‑19 risks; history of absenteeism from work due to COVID‑19; basic facts about COVID‑19; and prevention of COVID‑19. The data tools were administered by trained field research assistants using tablets running Open Data Kit (ODK) in Chichewa.

The primary outcome for this study was the harmonic mean number of confirmed (either by PCR or by repeat professional Ag-RDT) COVID‑19 cases in health care workers diagnosed within 5 days of symptoms onset. We hypothesized that there would be higher numbers of early COVID‑19 diagnosed in C19ST arm compared to the SOC arm. Secondary outcomes were comparisons of time off work, and reasons (e.g., any positive COVID‑19 tests or untested ‘flu like illnesses and any quarantine due to close contact). This data was captured at exit interview. An assessment of risk factors including vaccination history, and knowledge, attitudes, perceptions, and practices relating to COVID‑19 transmission and prevention was also explored and compared across facility testing-strategies.

### Data management and statistical analyses

Data quality assurance was implemented within the electronic form so that out-of-range values, inconsistent values and required variables were checked at the time of data collection. All tablets had full log-in details of the person collecting the data including a password. Access to the study database was protected by a password known only to the PI (AC) and the IT systems administrator. Data for study monitoring was periodically exported into comma separated values (CSV) from the study database on the MLW server for analysis and to raise plus resolve data queries.

Analysis was by intention-to-treat (ITT) using cluster-level summaries and a t-test, with adjustment for imbalance by fitting logistic regression models allowing the estimation of adjusted relative risks (RRs) and 95% CIs. In unadjusted analyses, a t-test was applied to the cluster-by-cluster estimates by trial arm. In adjusted analyses, a multiple random effects logistic regression model was fitted while excluding the trial arm in order to estimate residuals. A t-test then compared the residuals in place of actual estimates. Analyses used R and Stata 14.0 (Stata Corp, Texas, USA) [10]. Baseline characteristics were computed as proportions or median (interquartile range [IQR]) as appropriate. Estimates such as proportions and mean are reported along with 95% confidence intervals (CI).

## Results

**Fig 1:**
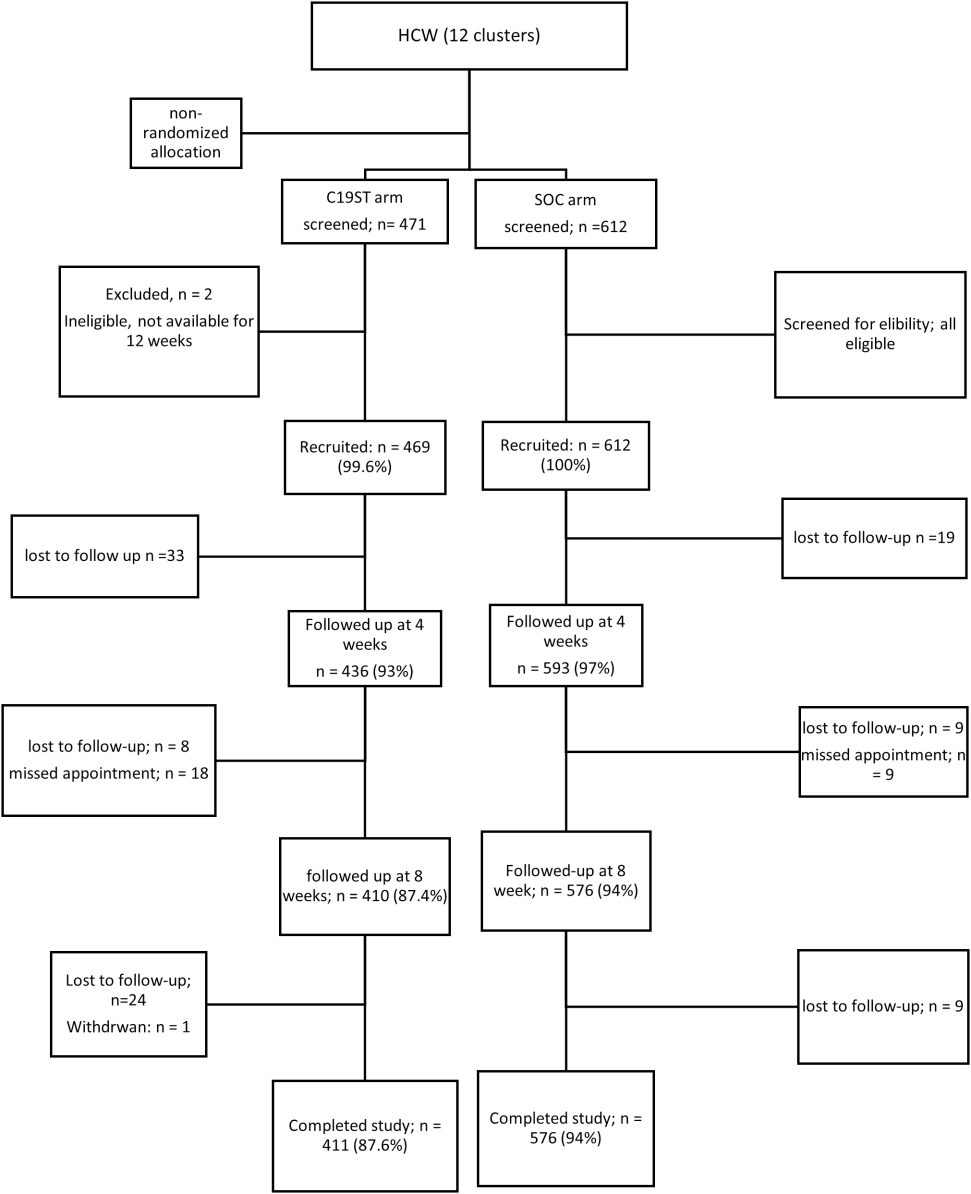
Flowchart of participants – follow-up and dropout of participant in C19ST and SOC arms.

Of 1081 participants recruited at baseline, 1012 (93.6%) completed 12 weeks follow-up (Fig 1).

There were 68 participants that were lost to follow-up and one participant who withdrew from the study. Participants in C19ST were slightly older (mean age 36.2 to 35.0 [p = 0.35]). The SOC arm had more participants from urban areas (74% to 66% [p = 0.006). There was a higher proportion of females in SOC (70%) (65% in C19ST, p = 0.056). Most of the participants reported being fully vaccinated against COVID‑19 (82% in SOC, 78% in C19ST [p = 0.150]) and not having any risk factors to severe COVID‑19 (69% in SOC and 77% in C19ST [p = 0.052]0(**Table 1**).

**Table 1:** Baseline characteristics of HCW by ARM.

Characteristic	N	Overall N = 1,081	C19ST, N = 469	SOC, N = 612	P-Value
**Age**, mean (SD)	1,081	35.5 (9.3)	36.2 (8.7)	35.0 (9.6)	0.035
**Sex**					
Female	1,081	732 (68%)	303 (65%)	429 (70%)	0.056
Male		349 (32%)	166 (35%)	183 (30%)	
**Vaccinated**					
No	1,081	319 (30%)	103 (22%)	113 (18%)	0.150
Yes		865 (80%)	366 (78%)	499 (82%)	
**Location**					
Rural	1,081	319 (30%)	159 (34%)	160 (26%)	0.006
Urban		762 (70%)	310 (66%)	452 (74%)	
**Work Experience**					
< 1 year	1,081	126 (12%)	39 (8.3%)	87 (14%)	0.002
1 – 5 years		388 (36%)	157 (33%)	231 (38%)	
5 – 10 years		189 (17%)	92 (20%)	97 (16%)	
> 10 years		378 (35%)	181 39%)	197 (32%)	
**Smoking**					
No	1,081	1,064 (98%)	456 (97%)	608 (99%)	0.006
Yes		13 (2.8%)	13 (2.8%)	4 (0.7%)	
**Body size**					
Normal	1,081	762 (70%)	328 (70%)	434 (70%)	0.837
Obese		6 (0.5%)	2 (0.4%)	4 (0.6%)	
Overweight		156 (14%)	67 (14%)	89 (15%)	
Underweight		157 (14%)	72 (15%)	85 (14%)	
Self-reported risk factors*					
None	1,081	781 (72%)	360 (77%)	412 (69%)	0.052
1-2		296 (27%)	108 (23%)	188 (31%)	
3-5		4 (0.4%)	1 (0.2%)	3 (0.4%)	

* Aged 60 or older; pregnant; having a condition that means you have a high risk of getting infections, for example HIV, taking medicine that can affect the immune system, for example, low doses of steroids (not counting ARVs); Having a lung condition, for example, asthma, chronic obstructive pulmonary disease, emphysema or bronchitis; having heart disease, for example, heart failure; having diabetes; having chronic kidney disease; waist circumference > 94 cm (males) or >80 cm (females); having liver disease, for example, hepatitis or cirrhosis

### Primary outcome: Geometric mean number of HCWs with a confirmed COVID‑19 diagnosis

Four participants in the C19ST self-tested positive, and all were confirmed positive on repeat testing using professional Ag-RDT, of which one was asymptomatic and three were symptomatic. In the SOC arm one symptomatic participant had a confirmed diagnosis of COVID‑19 using the professional Ag-RDT. Adjusted risk ratios and 95% confidence intervals were not done as the numbers were too small for meaningful comparison. The sample size calculation assumed harmonic mean number of 20 and 25 HCWs (standard deviation: 12) in SOC and the PCI arm confirmed (either by PCR or by repeat professional Ag-RDT) COVID‑19 diagnosed within three months, 6 clusters per arm of 100 HCWs each assuming intraclass correlation coefficient (ICC) of 0.05 would give 84.1% power to detect the stated difference in the harmonic mean.

### Secondary outcome: The number of days off due to suspected or confirmed COVID‑19 between the C19ST and SOC study arms

At first and second follow up, 128 (12.4%) and 109 (11.1%) reported having been ill in the previous 4 weeks respectively. Those that reported being sick at first and second follow-up and everyone (n = 971) who completed the study were asked if they took days off due to being sick. There was no difference in the proportion of people reporting taking time off between the 2 arms at 4-weeks (34.1% in SOC vs 34.5% in C19ST, p = 0.386) and at 8-weeks (35.8% in SOC and 28.6% in C19CT). However, at exit interviews (12 weeks), 9.5% in SOC arm reported taking time off and 2% in C19ST (p < 0.000) as shown in Table 2 below.

**Table 2:** The number of days off due to suspected or confirmed COVID‑19 between the C19ST and SOC study arms.

		C19ST		SOC		Overall		p-value
Time	Absent	n	%	n	%	n	%	
**Exit interviews (Week 12)**		n = 411		n = 560		N = 971		
	Yes	8	2.0	53	9.5	61	6.3	< 0.001
	No	403	98.0	507	90.5	910	93.7	
	Adjusted RR* (95% CI)	0.21 (0.08; 0.50)		1				< 0.001
**Second follow-up (Week 8)**		n = 28		n = 81		N = 109		
	Yes	8	28.6	29	35.8	37	34.0	0.486
	No	20	71.4	52	64.2	72	66.0	
	Adjusted RR* (95% CI)	0.80 (0.48; 1.33)		1				0.386
**First follow-up (week 4)**		n = 46		n = 82		N = 128		
	Yes	16	34.5	28	34.1	44	34.4	0.48
	No	30	65.5	54	65.9	84	65.6	
	Adjusted RR* (95% CI)	0.70 (0.50; 0.98)		1				0.386

C19ST: COVID‑19 self-testing; SOC: standard of care; RR: Risk ratio; CI: confidence interval; * Adjusted for clustering, age and sex.

### Secondary outcome: Usage of self-test kits

Among the HCW in C19ST arm who completed the study, 393 (83.8%) reported that they used the self-test kits twice weekly; 407 (86.7%) reported that they would use self-test kits in future is offered. There was a statistically significant differences in ease of testing between first test and repeat testing in the C19ST arm; 85.6% and 97.8% (p < 0.001) found testing easy or very easy on first and repeat testing respectively.

## Discussion

In this non-randomized trial, four HCW in C19ST arm and one in SOC were diagnosed with COVID‑19, while either asymptomatic or within five days of symptoms onset. Participants who self-tested positive had a confirmatory test by repeat professional Ag-RDT; received KN95 face masks and advised to isolate and continue with self-testing. Screening HCW for COVID‑19 using Ag-RDT was highly acceptable and was feasible among the participants in the C19ST arm. The proportion of HCW reporting regular twice weekly self-testing using the Ag-RDT test kits in the CT19ST arm was higher (83.8%) than what was reported by Heskin et al. in the United Kingdom (UK) (43.3%). Interest to use Ag-RDT self-test kits in future pandemics if given a chance was high in our study (86.7%) and in the UK study (94.5%). HCW also find self-testing quite easy to perform. In our study, 85.6% of the participants reported that they found self-testing easy or very easy on first testing and the proportion increased to 97.8%a with repeat testing (p < 0.001). Our findings are consistent with findings from the UK study which reported that 98.2% of the participants also found self-testing easy. Our results provide evidence that delivering COVID‑19 self-test kits to HCW through trained distributors located at a designated place within the hospital premises is feasible and highly acceptable at a time of low disease transmission. Although COVID‑19 incidence was low in the general population in Malawi, the risk was higher among HCW [11]. These results expand on the limited existing literature by generating evidence-based optimised delivery models for COVID‑19 self-testing in high-risk populations for future pandemic preparedness. The twice weekly self-testing model among hospital workers was also used in the UK, a setting with high transmission rate, and it enabled implementation of interventions to reduce staff-to-staff and staff-to-patient transmission [12].

In this present study, we also found that absenteeism from work due to COVID‑19 symptoms was statistically significant higher in SOC arm than in C19ST arm. This could suggest that routine screening may offer protection from unnecessary absenteeism. A likely explanation is that when HCW experience flu-like symptoms but self-test negative for COVID‑19, they were reassured about their risk of transmission, reducing the need for precautionary isolation and allowing them to continue working safely. However, the higher loss to follow-up observed in the C19ST arm in our study may have influenced the absenteeism findings, making it difficult to determine the precise impact of self-testing on work absenteeism. A study conducted in UK found that absenteeism from work among HCW was associated with psychological distress [13]. Other studies have suggested that having easy access to COVID‑19 helps to reduce anxiety related to the disease among HCW [14]. Therefore, routine screening for COVID‑19 has the potential to support optimal staffing levels in hospitals during pandemic waves, by reducing unnecessary quarantine and providing reassurance to staff experiencing mild or non-specific symptoms.

### Limitations

Limitations of our study include a nonrandomized design, and higher attrition in the C19ST arm (12.4%) than in SOC arm (5.9%) which might have introduced attrition bias. Therefore, the differences in the people who were lost to follow-up and those who continued with the study, might have caused the observed differences in study outcomes between the C19ST and SOC arms, and not the intervention itself, thereby overestimating the difference observed between arms [15]. ITT was used to minimize effects of the attrition bias for the primary outcome [16]. Furthermore, the study was only conducted in facilities from one district and at a time of low Sars-Cov- 2 transmission which yielded less positives making it difficult to conduct meaningful comparisons. The low count of confirmed COVID‑19 in our study was however not surprising as it was consistent with the local epidemiological trends at the time data was collected [17]. Additionally, 80% of the study population had reported that they were fully vaccinated against COVID‑19 hence the low incidence. Studies from different countries have shown that vaccine rates of at least 80% sustains reduction in confirmed COVID‑19 cases [18]. Although this study was conducted outside an active epidemic period, the implementation outcomes remain informative for future pandemic preparedness. While uptake and adherence may be higher during pandemic peaks, some contextual factor such as workload pressures, availability of resources, and local policies may differ during an outbreak. Therefore, the findings should be interpreted in consideration of these limitations.

## Conclusion

Given the substantial loss to follow-up in the intervention group and the smaller-than-expected number of outcome events in this non-randomized control trial, no definitive conclusions can be drawn regarding the primary outcome However, we found that COVID‑19 self-testing using Ag-RDTs was feasible to integrate for routine screening among HCW potentially reducing disruption to health services. This model has potential for wide scale up programmatically especially in resource-constrained settings. In the transition form an emergency response to long term disease management for COVID‑19, easy to use, quick and less resource demanding testing devices remain important for ongoing detection of emergent cases [19]. Although there were some study limitations, the adoption and use of Ag-RDT self-test kits for screening and diagnosis in high-risk populations should be considered not only for COVID‑19 but also for future pandemics. We therefore recommend further research on self-testing to generate additional evidence on effectiveness, usability, and integration into routine surveillance and emergency response systems.

## Supporting information

S1 Text HCW participation card. (DOCX)

S2 Text CONSORT Extension for Pragmatic Trials Checklist. (DOCX)

S3 Text Study Protocol. (PDF)

S1 Data Data file. (ZIP)

S1 Checklist Inclusivity in global research. (DOC)
