# Supplementary material for: COVID-19 diagnosis within five days of symptoms onset among healthcare workers in Malawi; Non-randomized control trial of self-testing using Ag-RDTs
Source: PLOS Glob Public Health. 2025 Dec 16;5(12):e0005604. doi: 10.1371/journal.pgph.0005604 (PMC12707653; doi:10.1371/journal.pgph.0005604)
Supplement: S1 Text — (DOCX) [file pgph.0005604.s001.docx]

**CHIPHASO CHAOTENGA MBALI MUKAFUKU WA 3ACP**

**
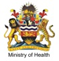

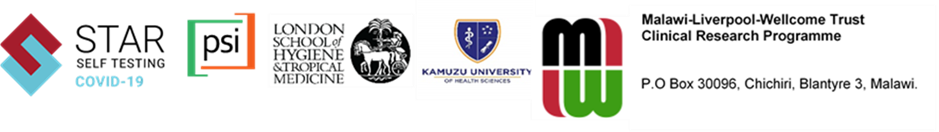
**

**3ACP Malawi: Enhancing access to COVID-19 tests**

|  |
| --- |

**Participant ID/barcode**

|  |
| --- |

**Site:**

| **Week** | **Date** | **Number of test kits** | **Issued by** | **Date of next visit** |
| --- | --- | --- | --- | --- |
| **1** |  |  |  |  |
| **2** |  |  |  |  |
| **3** |  |  |  |  |
| **4** |  |  |  |  |
| **5** |  |  |  |  |
| **6** |  |  |  |  |
| **7** |  |  |  |  |
| **8** |  |  |  |  |
| **9** |  |  |  |  |
| **10** |  |  |  |  |
| **11** |  |  |  |  |
| **12** |  |  |  |  |

**notes**

1. **participant should collect self-test kits every week /otenga mbali akuyenela kuzatenga zipangizo zoziyezera sabata iliyonse**
2. **Participant should respond to study questionnaires in week 1, 4, 8 and 12/ otenga mbali akuyenela kusunsidwa mafunso akafuku fuku musabata 1, 4, 8 ndi 12**
