# Supplementary material for: COVID-19 diagnosis within five days of symptoms onset among healthcare workers in Malawi; Non-randomized control trial of self-testing using Ag-RDTs
Source: PLOS Glob Public Health. 2025 Dec 16;5(12):e0005604. doi: 10.1371/journal.pgph.0005604 (PMC12707653; doi:10.1371/journal.pgph.0005604)
Supplement: S3 Text — (PDF) [file pgph.0005604.s003.pdf]

3ACP Malawi: Enhancing access to COVID-19 tests

Africa, Asia, Americas COVID-19 Preparedness (3ACP)

Malawi Investigators
Dr Augustine Talumba Choko (PhD)[1]: Principal Investigator
Mr Richard Chilongosi (MPH)[2]: Implementation
Dr Peter MacPherson (PhD)[1, 4]: Epidemiological input
Ms Linda Sande (MSc)[1]: Health Economics
Dr Nicola Desmond[1, 4]: Social Science Lead
Ministry of Health (MoH), Malawi
Mr. Godfrey Kadewere (BSc)[5]: National guidance
International Collaborators:
Prof Elizabeth Corbett (PhD)[1, 3]: Chief Investigator and 3ACP Research Director
Dr Karin Hatzold (MSc)[6]: 3ACP Project Director
Prof Katherine Fielding (PhD)[3]: Statistical and epidemiological input
Prof Anna Vassall (PhD)[3]: Economist
Prof Mark Jit (PhD)[3]: Mathematical modeller
Collaborating Institutions:
1. Malawi Liverpool Wellcome Trust Clinical Research Programme (MLW), Blantyre, Malawi
2. Population Services International, Malawi Office
3. London School of Hygiene and Tropical Medicine (LSHTM), London, United Kingdom
4. Liverpool School of Tropical Medicine, Liverpool, United Kingdom
5. Ministry of Health: Diagnostics
6. Population Services International, RSA

Funder: Unitaid

Sponsor: London School of Hygiene & Tropical Medicine is the main research sponsor for this study. For further information regarding the sponsorship conditions, please contact the Research Governance and Ethics Office:

London School of Hygiene & Tropical Medicine; Keppel Street, London, WC1E 7HT, +44 (0) 20 7636 8636.

This study will adhere to the principles outlined in the International Council for Harmonisation Good Clinical Practice (ICH GCP) guidelines, protocol and all applicable local regulations.

Version 4.0 **last amended 11 Nov 2022 by ATC**
Signature: **Date: 11 Nov 2022**

Table of Contents

Acronyms 4

1. Executive summary 5

2. Background 7

2.1. Introduction 7

2.2. Self-testing using COVID-19 Ag-RDTs 8

2.3. Economics of COVID-19 Ag-RDT testing and self-testing 9

2.4. Feasibility and usability of self-testing and then optimizing performance of Ag-RDTs in general through various approaches and strategies 9

2.5. Background studies leading to the project 9

2.6. Current COVID-19 status in Malawi 10

2.7. Policy impact – intentions of this protocol 11

3. Study rationale 12

4. Objectives of the study 12

4.1 Broad objective 12

4.2 Specific objectives 12

5. Methods 12

5.1. Type of research study 12

5.1.1. A non-randomized controlled study among health care workers 14

5.1.2. Routine provider-delivered diagnostic COVID-19 Ag-RDT testing in out-patients 19

5.1.3. Investigating potential COVID-19 outbreaks in congregate, populated settings 22

5.1.4. Secondary distribution of self-test kits 25

5.2. Study place 29

5.3. Study population 30

5.4. Qualitative process evaluation 31

5.5. Costing and cost-effectiveness 33

5.6. Clinical aspects and PPE 34

5.7. Study period 34

5.8. Overall sample size considerations 35

5.9. Data collection 35

5.10. Data management and analysis 37

5.10.1. Data management 37

5.10.2. Statistical analysis 38

5.10.3. Qualitative data analysis 38

5.11. Results presentation 39
5.12. Dissemination of results 39
6. Overall ethical considerations 39
6.1. Ethics approval 39
6.2. Written or witnessed informed consent 40
7. Anticipated benefits and harms 41
8. Conflict of interest 42
9. Compensation for participants 42
10. Possible constraints 42
11. Requirements 43
11.1. Personnel 43
11.2. Materials and consumables 43
11.3. Equipment 43
11.4. Space 43
11.5. Miscellaneous 43
12. Budgetary estimates 44
13. Justification of budget 44
14. References 45
15. List of Appendices 48

Acronyms

ANS	Anterior nasal swab
COMREC	College of Medicine Research Ethics Committee
COVID-19	Coronavirus Disease 2019
COVID-19 Ag RDT	Coronavirus Disease 2019 antigen rapid diagnostic test
DEC	District Executive Committee
DHMT	District Health Management Team
EC	Ethics Committee
FIND	Foundation for Innovative new Diagnostics
GCP	Good Clinical Practice
HCW	Health Care Worker
HEC	Health and Environment Committee
HICs	High Income Countries
HIV	Human Immunodeficiency Virus
ICC	Intraclass Correlation Coefficient
IFU	Instructions for Use
IRB	Institutional Review Board
KUHeS	Kamuzu University of Health Sciences
LMICs	Low- and Middle-Income Countries
LSHTM	London School of Hygiene and Tropical Medicine
MLW	Malawi Liverpool Wellcome Clinical Research Programme
MoH	Ministry of Health
NAAT	Nucleic acid amplification test
ODK	Open Data Kit
OPD	Out-Patient Department
PCI	Programmatic COVID-19 interventions
PCR	Polymerase Chain Reaction
PHC	Primary Health Centre
POC	Point-of-care
PPE	Personal Protective Equipment
PSI	Population Services International
RDT	Rapid diagnostic tests
RT-PCR	Real-Time Polymerase Chain Reaction
SARS-CoV-2	Severe Acute Respiratory Syndrome Coronavirus 2
SOC	Standard of Care
TIDIeR	Template for Intervention Description and Replication
WHO	World Health Organization

1. Executive summary

Type of study: We wish to conduct quantitative and qualitative evaluations of COVID-19 Antigen rapid diagnostic testing (RDT) strategies (COVID-19 Ag-RDT) including self-testing and linkage to treatment and prevention.

Problem: Testing, isolation, contact tracing and personal preventive equipment remain key to managing the spread of COVID-19 infection. NAAT are required for diagnosis although antigen rapid diagnostic tests (Ag-RDTs) provide a quicker and instrument-free alternative. Many resource-constrained settings face shortages of testing supplies, skilled laboratory personnel, high costs and logistical challenges to implement NAAT.

Broad objective: To evaluate uptake and feasibility of implementing COVID-19 Antigen RDT (COVID-19 Ag-RDT) strategies including self-testing.

Specific objectives

1. To compare the number of confirmed COVID-19 cases **diagnosed within five days of symptoms onset** in health care workers between the 6 intervention primary care clinics offering unsupervised **serial twice-weekly self-testing** to screen for asymptomatic SARS-CoV-2 and 6 standard of care ones.
2. To compare **the number of days off among health workers due to suspected or confirmed COVID-19** between the intervention and the standard of care arms.
3. To evaluate the acceptability of professional use Ag RDT in congregate settings and outpatients.
4. To evaluate the acceptability of KN95 masks by the COVID-19 positive participants to prevent infection of contacts in congregate settings and outpatients
5. To evaluate the acceptability of unsupervised **self-testing** provided **through secondary distribution of COVID-19 Ag-RDT self-test kits** to contacts of patients testing positive for COVID-19 by building on optimisation studies already conducted in Malawi.
6. To evaluate qualitative views regarding problems or challenges faced by travellers with respect to COVID-19 testing, isolation for those testing positive and/or care for those with complications.
7. To conduct costing and cost effectiveness analysis of the implementation models of COVID-19 testing for the different use cases: self-testing and professional Ag-RDT.

Methodology

Designs, settings and participants

We will use a combination of prospective studies including cohort, cross-sectional and process evaluations to evaluate key components of the five use cases. For the repeat use case among health care workers (HCWs), we will provide 2 self-test kits per week in line with international best practice. ACON Flowflex self-tests will be used for all self-testing research purposes . A cross-sectional study design will be employed for the OPD attendees from Blantyre health facilities. For the workplace and congregate settings, we will provide access to POC Ag-RDTs in a cross-sectional design.

Main sample size considerations: For the health care worker component: With an assumed harmonic mean number of 20 and 35 HCWs (common standard deviation: 12) in SOC and the PCI arm confirmed (either by PCR or by repeat professional Ag-RDT) COVID-19 diagnosed within three months, 6 clusters per arm of ~100 HCWs each, the study will have 84.1% power to detect the stated difference of harmonic count of 15. For studies offering self-testing, we conservatively assume that 60% of participants will be willing to self-test for COVID-19 if offered. For the sample proportion to

be estimated to within +/-0.05 (5%) using the 95% confidence level, a sample of 370 participants would be required.

Expected findings and dissemination: The results will be used to inform MoH on COVID-19 scale-up plans and will also be disseminated through Kamuzu University of Health Sciences including COMREC, and through conference presentations and publication in peer-reviewed journals.

2. Background

2.1. Introduction

COVID-19 caused an estimated 18.2 million excess deaths, globally, by December 2021 including 5.9 million reported COVID-19 deaths.(1) By July 2022 over 570 million infections had been reported globally with over 6.3 million reported deaths (2, 3) although with much higher cumulative infections (3.4 billion) and mortality estimated from analysis of serosurveys and excess mortality trends the pandemic.(4)

Proportion to population, sub-Saharan Africa has much lower SARS-CoV-2 test uptake and far fewer patients diagnosed or hospitalized than Europe and North America, but extremely high prevalence of infection with 70.5% of all Africans estimated to have had at least one SARS-CoV-2 infection by end 2021, even before Omicron. Illustrating the profound testing gap in Africa, only an estimated 7 per 1000 infections were diagnosed and reported – with no trends towards this increasing over time. This suggests that most African countries have still not developed effective testing systems or strategies. In contrast an estimated 44.6% of all infections in high income countries in 2020-21 were diagnosed and reported.

With highly effective vaccines and oral antivirals now available (5) plus substantial population immunity to severe outcomes, and the emergence of highly transmissible but less pathogenic Omicron strains, testing programs globally have downscaled towards more targeted strategies aimed at infection-prevention in the highest risk settings and early diagnosis and outpatient antivirals for the highest risk individuals (6). In Africa, there is still need to establish and evaluate key use cases based on rapid diagnostic testing to establish acceptability and reach to high risk individuals eligible for oral antivirals.

A number of different diagnostic test systems have met accuracy standards set by World Health Organization and other Strict Regulatory Authorities. The most sensitive diagnosis of acute SARS-Cov-2 infection is provided by real-time polymerase chain reaction (RT-PCR) and other nucleic acid amplification tests (NAAT).(7) However, in many low resource settings NAATs can only be performed by skilled personnel in centralized facilities resulting in critical access barriers and long turnaround times. Shortages of testing supplies, requirements for skilled laboratory personnel, high costs and logistical challenges often mean that demand for tests exceeds supply. These challenges have been encountered globally but are most pressing in low and middle-income countries and especially in sub-Saharan Africa where per capita rates of testing (adjusted for population size) are a small fraction of the numbers of tests conducted in other regions. Capacity to meet testing demand using NAAT remains well below true needs, with long distances to the nearest laboratory, limited scope for free testing, and high user charges for private laboratory testing each presenting critical barriers to scale-up. Long turnaround times may also limit the clinical utility of NAAT results.

Device-free point-of-care (POC) tests can provide timely SARS-Cov-2 diagnosis, contributing to optimized clinical management and prevention efforts as well as to timely identification of new outbreaks, potentially representing new variants.(8) As access to novel therapeutics becomes more widespread, considerations of timely access to early diagnosis will become even more critical as antiviral agents are most effective if taken within 5 days of symptom onset.

Lateral flow assays that directly detect SARS-CoV-2 antigens (antigen rapid diagnostic tests, or Ag-RDTs) present when the virus is actively replicating in the nose and throat have proved to have high clinical utility, with 10s of millions of tests used each week as part of the response to the Omicron

surge. Ag-RDTs provide results within 15-20 minutes after the collection of an upper respiratory swab sample, typically an anterior nasal swab (ANS) without need for expensive equipment. These have been recommended by World Health Organization to complement NAAT since 2020, with the October 2021 updated Interim Guidelines including a wider range of use cases with less emphasis on need to confirm positive results.(9, 10) Because Ag-RDTs detect virus proteins (not nucleic acid) they correlate closely with infectiousness (11), including early asymptomatic infection, and their low cost and ease of deployment makes a 2-test serial testing strategy (typically on days 2 and days 5 to 7 post-exposure) feasible, effective and affordable for **contact tracing** including “test-for-release” aiming to minimize need for time off work after exposure. However, overall diagnostic sensitivity is lower than NAAT, notably so after day 7 of symptom onset.

2.2. Self-testing using COVID-19 Ag-RDTs

COVID-19 self-testing was strongly recommended by the World Health Organization (WHO) in March 2022 as an additional strategy to complement professionally administered testing services.(12) Self-testing, has been successfully used in other disease areas such as HIV where uptake is ubiquitously high including among hard to reach and key populations.(13-15) In general, COVID-19 self-testing has the potential to increase diagnostic capacity for COVID-19 and reduce access barriers as well as prevailing inequalities due to ease of distribution and being extremely convenient.(16) However, COVID-19 self-testing has so far been widely implemented and made available in high income countries.(16-18) As with HIV self-testing, lack of linkage for next steps with COVID-19 is a potential concern due to stigma, loss of economic opportunities due to isolation implications, and fear of complications including death.

Gathering evidence from multiple settings is important: we have showed a marked difference in unassisted HIV self-test performance between rural and urban settings in Southern Africa.(13) This type of problem can be identified with techniques such as cognitive interviewing and pre-empted with in-person demonstration or video clips (19). This protocol will build on the previous COVID-19 work in Malawi to support ANS self-testing in health workers, supporting optimization of scale-up materials to support self-testing by intended-users in their intended-settings.(20)

Testing for treatment of COVID-19 in Africa

Outpatient antiviral treatment requires prompt diagnosis of infection and risk-based management of COVID-19 in patients at the highest risk of hospitalization. The current regimen of choice is a 5 day oral course of nirmatrelvir-ritonavir (Paxlovid) (5). Prompt diagnosis is critical as oral antivirals are most effective and can reduce subsequent hospitalization or death by over 80% if initiated within 5 days of the onset of symptoms, before serious manifestations have developed (21). This means that antiviral treatment decisions need to be based on assessment of key clinical vulnerabilities, such as advanced HIV disease, rather than disease severity (5). Testing-for-treatment is then most readily supported by rapid diagnostics tests.

2.3. Economics of COVID-19 Ag-RDT testing and self-testing

The need to consider rational use of resources will be addressed in the current protocol by costing, consideration of service provision efficiencies and estimation of prevention and care impacts to allow incremental cost-effectiveness to be compared against no alternative testing, NAAT testing, and to compare self-test with professional Ag-RDT alternatives.(22) Data on costs and cost-effectiveness of self-testing use cases remain sparse. Costs of accessing testing for COVID-19 in low-resource settings are also limited. The costing component in this protocol will address this data scarcity by evaluating costs of providing and accessing self-testing in comparison to no alternative testing, NAAT testing, and to compare self-test with professional Ag-RDT alternatives. Service integration where self-testing is integrated with Ag-RDT, can also be associated with high time burden on existing staff (23). Understanding such time commitments and considering different varying degree of integration will also be addressed within the protocol.

2.4. Feasibility and usability of self-testing and then optimizing performance of Ag-RDTs in general through various approaches and strategies

Diagnostic tools that can increase peak testing capacity reduce the user costs and turnaround-time for COVID-19 infection detection are needed to optimize clinical management and prevention efforts. Such tools include the development and evaluation of novel tests and the implementation of already tried and tested tools.(10) Among innovative strategies of COVID-19 testing is self-testing which is already under implementation in high income countries.(17)In Malawi at present, Ag-RDT is used for diagnosis of COVID-19 in cases where NAAT is unavailable or where prolonged turnaround times preclude clinical utility. COVID-19 self-testing has the real potential to complement standard testing and to significantly reduce the stress on depleted health care providers.(24) Indeed, COVID-19 itself may lead to significant number of key personnel being absent from work or the sheer volumes of demand for COVID-19 testing and care may run thin the service delivery thereby impeding efforts.

Ag-RDT tests that directly detect SARS-CoV-2 antigens are now becoming widely available in High Income Countries (HICs) and not yet in Low and Middle Income Countries (LMICs) and have the advantage of producing results within 15-20 minutes compared to NAAT based testing which can take up to 5 days.(25, 26) A key issue with these Ag-RDTs is their potential sub-optimal accuracy compared to NAAT although there are now Ag-RDTs that can get to 97% sensitivity of infectious cases, based on the relationship between viral load and infectiousness.(8, 27)

2.5. Background studies leading to the project

Choko, the Malawi PI of this study, recently completed one of the first studies investigating the feasibility of self-sampling and self-testing for COVID-19 in low-income countries, supported by Foundation for Innovative New Diagnostics (FIND). Following extensive work on contextualizing the instructions for use (IFUs), we found that our study participants were generally able to conduct their own sampling of the AN and also were able to self-test for COVID-19. During the self-testing work which coincided with the 4th wave, we had some interesting observations with respect to people's choices and decision-making when they self-test positive and how that might impact onward

transmission of COVID-19. The study showed that there is need for more work around Personal Protective Equipment (PPE) particularly high-quality mask (e.g. N95 and KN95) wearing and health seeking for those who self-test positive for COVID-19. Subsequently, this study was one of the first studies in low- and middle-income countries that informed the interim guidelines for the WHO that strongly recommended COVID-19 self-testing.(12)

2.6. Current COVID-19 status in Malawi

The World Health Organization (WHO) recommends sensitivity of ≥80% and specificity of ≥97% for Ag-RDTs (28). PanBio with sensitivity of 85.5% (95% CI: 78.0–91.2) and specificity of 100.0% (95% CI: 99.1–100), and STANDARD Q Ag-RDT with sensitivity of 89.0% (95%CI: 83.7–93.1) and specificity of 99.7% (95%CI: 98.4–100) for professional use are approved and already in use in Malawi (29) , and we will use these two interchangeably as per availability at the time of testing. The project will use ACON Flow*flex*™ COVID-19 Antigen Home Test for self-testing with clinical sensitivity of 92.1% (82.7, 96.6) and specificity of 99.5% (97.5, 99.9) (30). ACON Flowflex is not approved in Malawi for self-testing. However, ACON Flowflex is FDA approved and we will be using it under the research mark for the purposes of the study. COVID-19 self-testing is not (yet) supported by Malawi policy guidelines. Thus, the data that our project will generate will be critical informing policy decisions to support COVID-19 self-testing in Malawi. Assessment of the efficacy of the tests is not an objective of this study. The ACON Flowflex test kit is not currently registered or used for professional or self-testing in Malawi. At a meeting held on 9th June with the Ministry of Health As technical working group we presented the ACON Flowflex kit and the Ministry agreed to conduct in-country validation. Thus, a waiver was obtained from the Malawi Government to use the ACON flowflex test kit for research use only.

Currently, the Government and major donors are procuring the professional use versions of the PanBio and STANDARD Q test kits. These test kits are also available for purchase in some pharmacies in country.

The current COVID-19 testing in Malawi is symptom-based starting with professional Ag-RDTs or as per high need such as for travel purposes in which case PCR testing is required. All positive RDT results are not confirmed, if COVID-19 symptoms persist following negative Ag-RDT testing PCR is done to confirm the negative Ag RDT result. PCR is only available in very selected often high throughput or reference laboratories or hospitals. However, Ag-RDTs are available and offered to out-patients in most clinics including some primary care clinics within Malawi. There is no routine screening of health care workers (HCWs) in Malawi although COVID-19 testing is readily available for HCWs who are symptomatic or those who wish to be screened.

Current Malawi COVID-19 government processes as of September 2022(31)

a) Testing and follow up of symptomatic people: tested using professional Ag-RDT. If test is positive treat as having confirmed COVID-19 and manage accordingly. If test is negative, perform real time PCR and manage accordingly.
b) Testing and follow up of asymptomatic people entering health care clinics: Asymptomatic COVID-19 contacts (and high risk populations such as health care workers). Tested using professional Ag-RDT. If test is positive isolate and observe social prevention measures. Perform real time PCR if available. If test is negative, manage as negative.

c) Screening at borders: Treated as travellers under asymptomatic non-contacts and outbreak investigation. Tested using professional Ag-RDT. If test is positive treat as having confirmed COVID-19 and manage accordingly. If test is negative, treat as negative. Additionally, all travellers out of Malawi must either have a negative RT-PCR negative result within 72 hours of travel or evidence of valid full vaccination.
d) Referral of self-test-positive participants to PCR testing: No explit guidance exists.
e) Follow up and any mandatory restrictions of people testing positive by RDTs: All people with confirmed positive COVID-19 to isolate for 10 days. After the isolation period, tested using professional Ag-RDT. If test is positive treat as having confirmed COVID-19 and manage accordingly. If test is negative, treat as negative.
f) Follow up and any mandatory restrictions of people confirmed by PCR: All people with confirmed positive COVID-19 by RT-PCR to isolate for 10 days. After the isolation period, tested using professional Ag-RDT. If test is positive treat as having confirmed COVID-19 and manage accordingly. If test is negative, treat as negative.
g) Contact tracing (32): All close contacts should quarantine for 10 days from the day of the person testing positive. If any close contact / family member develops any symptoms it is not necessary for them to be tested as well. They should be treated as if they are positive.

Antivirals are not yet available in Malawi, but are being introduced to 5 treatment centers through a Unitaid-sponsored initiative. Treatment eligibility criteria have not yet been finalized at the time of writing (Sept 2022).

2.7. Policy impact – intentions of this protocol

The Malawian government identified as priority the use-cases detailed in this protocol to support the scale up and development of professional use- and self-testing models in Malawi. Roll-out of professional-use RDTs is not well-established in Malawi, despite its inclusion in national policy, and this research therefore will highlight areas of best practice that can be cascaded through the Technical Working Group within the Ministry of Health (MOH) to national providers. The evaluation of self-testing models, i.e., routine screening of high-risk occupations, and secondary distribution models for contacts will answer existing gaps in national COVID-19 diagnostic services on affordable ways to reach and target individuals who may benefit from antivirals due to their age and comorbidities with early diagnosis, and affordable ways to increase peak testing capacity and provide contact tracing more effectively in the event that Malawi experiences a further wave of severe COVID such as seen during the Beta and Delta variant waves. In addition, the current national policy guideline which treats all contacts as assumed positive, and requiring isolation, was implemented due to the labor and resource-intensive nature of inviting contacts for professional Ag-RDTs or PCR. The proposed approach in this study therefore which offers COVID-19 self-tests to contacts, (and which will be fully costed) provides evidence as to whether a more nuanced contact tracing approach can be considered for national policy.

To date, this work has supported the formation of Technical Working Groups on RDT and self-testing within the Ministry to draft guidelines for potential scale up and implementation with technical support and representation from both PSI and MLW.

We are working with the following officials from the Ministry of Health in Malawi:
represented on those

1. Mr. Godfrey Kadewere, Director, HTSS-Diagnostics, MoH

2. Dr. Gift Kawalazila, Director of Health and Social Services, Blantyre DHO
3. Dr. Irene Zuze, Director of Health and Social Services, Mwanza DHO
4. Dr. Isaac Mbingwani, Director of Health and Social Services, Dedza DHO
5. Dr. Juliana Kanyengambeta, Director of Health and Social Services, Mchinji DHO

3. Study rationale

Large volume demand for COVID-19 testing and generally the demand for COVID-19 testing increases with the emergence of every new variant. Thus, decentralized testing that expands access and enables people to participate in testing at their convenience is urgently needed, particularly in resource-constrained settings such as Malawi. The study seeks to explore the impact of Ag-RDTs on increasing the uptake of testing in various use cases and for detecting outbreaks as well as incidence including the introduction of self-testing. Data on many of these aspects remain scant, especially in low-income countries. Furthermore, additional insights are needed on whether or not usability and feasibility of self-testing for COVID-19 improves with multiple as opposed to single self-testing. Note that specific policy implications are presented and discussed section 2.7.

4. Objectives of the study

4.1 Broad objective

To evaluate uptake and feasibility of implementing COVID-19 Antigen RDT (COVID-19 Ag-RDT) strategies including self-testing. Evaluation will be in different use cases: *health care workers; outbreak Investigation; testing of OPD attendees and contact tracing in Malawi.*

4.2 Specific objectives

The specific objectives are specified below.

1. To compare the number of confirmed COVID-19 cases **diagnosed within five days of symptoms onset** in health care workers between the 6 intervention primary care clinics offering unsupervised **serial twice-weekly self-testing** to screen for asymptomatic SARS-CoV-2 and 6 standard of care ones.
2. To compare **the number of days off among health workers due to suspected or confirmed COVID-19** between the intervention and the standard of care arms.
3. To evaluate the acceptability of professional use Ag RDT in congregate settings and outpatients.
4. To evaluate the acceptability of KN95 masks by the COVID-19 positive participant to prevent infection of contacts in congregate settings and outpatients
5. To evaluate the acceptability of unsupervised **self-testing** provided **through secondary distribution of COVID-19 Ag-RDT self-test kits** to contacts of patients testing positive for COVID-19 by building on optimisation studies already conducted in Malawi.
6. To evaluate qualitative views regarding problems or challenges faced by travellers with respect to COVID-19 testing, isolation for those testing positive and/or care for those with complications.
7. To conduct costing and cost effectiveness analysis of the implementation models

5. Methods

5.1. Type of research study

We wish to conduct quantitative and qualitative evaluations of COVID-19 Antigen rapid diagnostic testing (RDT) strategies (COVID-19 Ag-RDT) including self-testing and linkage to treatment and

prevention in different use cases. These are: *health care workers; outbreak Investigation; testing of out-patient department (OPD) attendees and contact tracing.* The specific methodology is presented under each of the following use cases that are also described in summary in Table 1:

Table 1: Summary of project components

				**Research by MLW**	**Implement by PSI**
**Use-case**	**Study design and activity type**	**Main outcome**	**Component study activities**	**Sample size***	**Target number of test kits***
1. Health care workers	*Self-testing:* Non-randomised 2-arm trial	Harmonic mean number of health care workers with confirmed COVID-19 infection between study arms	i. Epi interviews establishing participant sociodemographic profiles<br>ii. Sub-sample qualitative interviews establishing acceptability of process<br>iii. No costing activities	12 clusters; N=1200<br><br>Of which;<br>**All** eligibility assessed<br>i. 1200 baseline epi interviews, and exit interviews (+follow-up of all positives)<br>ii. Subsample of 20 qualitative interviews, from intervention arm.	7,200 self-tests (600 in trial arm, testing twice weekly for 12weeks.)
2. Congregate settings	*Professional testing:* Descriptive process evaluation	Proportion tested for COVID-19 (uptake of professional tests)	i. Epi interviews establishing participant sociodemographic profiles<br>ii. Sub-sample qualitative interviews establishing acceptability of process<br>iii. Sub-sample participant cost interviews, and provider cost observations establishing cost of process	323 participants<br><br>Of which;<br>**All** eligibility assessed<br>i. 323 epi interviews<br>ii. subsample of 20 qualitative interviews (5 per outreach site)<br>iii. subsample of 20 patient-level costing interviews and 12 provider observations	5000 professional-use tests
3. Outpatients	*Professional testing:* Descriptive process evaluation	Proportion tested for COVID-19 (uptake of professional tests)	i. Epi interviews establishing participant sociodemographic profiles<br>ii. Sub-sample qualitative interviews establishing acceptability of process	323 participants<br>**All** eligibility assessed<br>i. 323 epi interviews<br>ii. subsample of 20 qualitative interviews (5 per outreach site)<br>iii. subsample of 20 patient-level costing interviews and 12 provider observations	10,600 professional-use tests

			iii. Sub-sample participant cost interviews, and provider cost observations establishing cost of process		
4. Secondary distribution of self-test kits	*Self-testing:* Demonstration project evaluation	Proportion of contacts self-tested for COVID19 (contact uptake of self-test kits)	i. Epi interviews establishing participant sociodemographic profiles<br>ii. Sub-sample qualitative interviews establishing acceptability of demonstration model<br>iii. Sub-sample participant cost interviews establishing cost of model *(no provider observations)*	Any positive participant from the use-cases.<br>**All** eligibility assessed<br>i. **All** receive baseline epi interview, and telephone follow up after 14 days<br>ii. subsample of 20 qualitative interviews<br>iii. subsample of 20 patient-level costing interviews<br><br>Minimum requirement of 370 contacts to ascertain uptake[i]	6,000 self-tests

MLW: Malawi Liverpool Wellcome Trust Clinical Research Programme; PSI: Population Services International

5.1.1. A non-randomized controlled study among health care workers

Objectives

To compare the number of confirmed COVID-19 cases diagnosed within 5 days of symptoms onset, and the number of days off due to suspected or confirmed COVID-19 between the programmatic COVID19 intervention (PCI) and standard of care (SOC) study arms.

To increase early diagnosis and linkage to care in the self-testing facilities compared to a) pre-intervention rates through retrospective data extraction from HCW data from the facilities and prospective interviews with HCWs and b) between the two different testing strategies.

Outcomes

The primary outcome for this component is the harmonic mean number of confirmed (either by PCR or by repeat professional Ag-RDT) COVID-19 cases in health care workers diagnosed within 5 days of symptoms onset. We hypothesize that there will be higher numbers of early COVID-19 diagnosed in PCI arm compared to the SOC arm.

Secondary outcomes are comparisons of time off work, and reasons (e.g., any positive COVID-19 tests or untested 'flu like illnesses and any quarantine due to close contact). This data will be captured at exit interview.

An assessment of risk factors including vaccination history, and knowledge, attitudes, perceptions, and practices relating to COVID-19 transmission and prevention will also be explored and compared across facility testing-strategies.

Qualitative interviews with a subsample of healthcare workers in the self-testing arm will establish acceptability of the intervention.

Overview

We will conduct a non-randomized controlled study among health care workers (HCWs) in urban and rural Blantyre comparing two strategies delivered to 12 facilities. Health workers at six self-testing health facilities (PCI arm), will be offered twice-weekly self-testing for early diagnosis and infection prevention control, in addition to occupational diagnostic COVID-19 testing with COVID-19 Ag-RDTs that will be introduced to all 12 facilities under enhanced standard of care (SOC) (Figure 1).

Figure 1: Schema of the health care worker study design

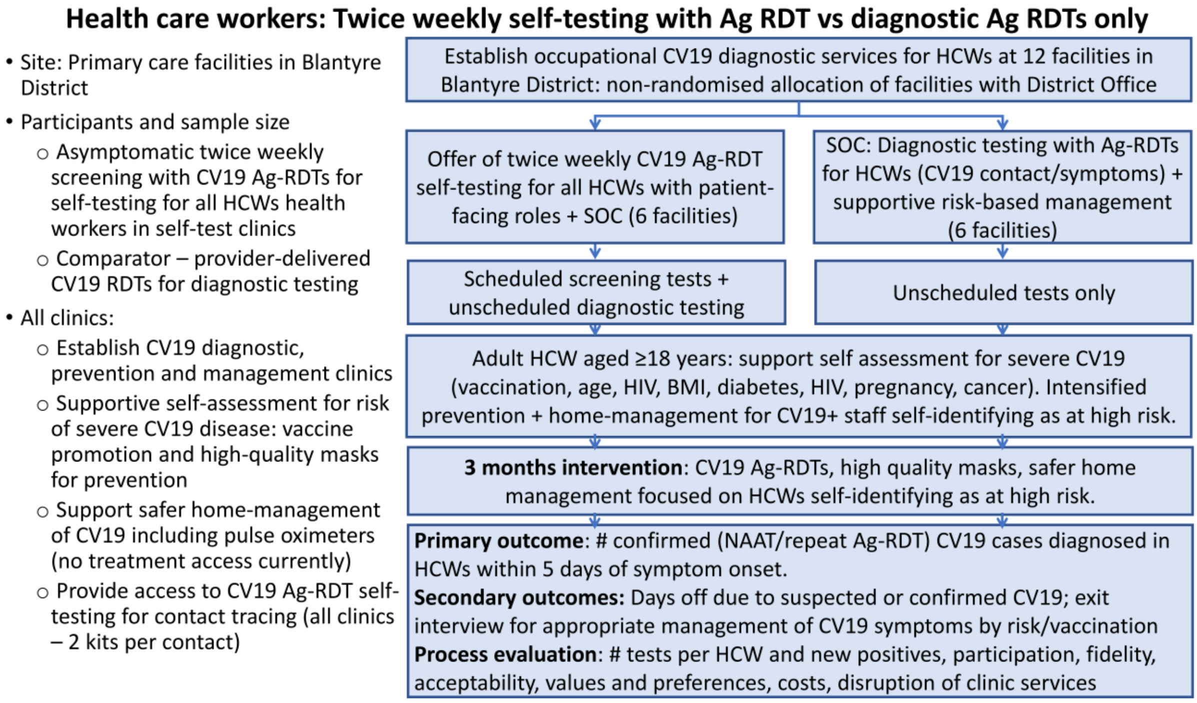

The criteria for allocating health facilities to the intervention and the standard of care arms was based on location (urban, peri-urban and rural) as well as the size of the health facility.

All HCWs (including administrators, maintenance staff, support staff, and clinicians) working in the targeted 12 health facilities of Blantyre District will be offered the opportunity to participate in the study. Enhanced routine diagnostic testing for staff, provision of high quality KN95 masks and health education to promote self-assessment of risk factors for severe COVID-19 and the benefits of vaccination will be offered to HCWs in all 12 facilities during the period of the study. In the intervention facilities, participating HCWs will each receive two COVID-19 Ag-RDT self-test kits per week for 12 weeks from Population Services International (PSI). PSI will be responsible for all implementation components, including training and provision of kits and masks.

All HCWs who self-test positive will be asked to confirm infection with anterior nasal swab for PCR or repeat professional use Ag-RDT and will receive high quality masks for their contacts as well as themselves. They will be prioritized for COVID-19 care and treatment based on severity of illness and presence of self-assessed risk factors. We will assess the proportion of health care workers who are linked to care by PSI as part of the study interventions and will compare this proportion between the intervention and the standard of care health facilities.

Activities:

Epidemiological

1200 healthcare workers (600 in each trial arm) will receive a structured questionnaire (CH01A) to collect data on the participants' demographic characteristics, assess their risk for severe COVID-19, knowledge on basic facts about COVID-19 and their practices and behaviours regarding COVID-19 prevention at baseline in both study arms.

Another structured questionnaire (CH02) shall be used to follow-up the participants monthly over the 12 weeks to find out if they had any COVID-19 symptoms over the past four weeks. For those who had the symptoms, we will ask about the management and care they received.

After the three months follow-up period, a structured questionnaire (CH03) shall be used to conduct an exit interview for all participants where we reassess the participant's risk for severe COVID-19, knowledge on basic facts about COVID-19, absenteeism from work due to COVID-19, their practices and behaviours regarding COVID-19 prevention, COVID-19 related stigma and discrimination, and experiences with COVID-19 self-testing.

Qualitative

A subsample of 20 participants will be selected from the intervention arm to ascertain the acceptability of the trial in line with Proctor's implementation outcomes (Clients SSI topic guide English).

Study coordinators and 2 study providers will also be interviewed to write up the TIDieR description for this use-case, as well as to input into the acceptability of the use-case in line with Proctor's implementation outcomes (Provider SSI topic guide & CK01).

Cost and cost-effectiveness

There is no costing work in this use-case.

Sample size considerations

With an assumed harmonic mean number of 20 and 35 HCWs (common standard deviation: 12) in SOC and the PCI arm confirmed (either by PCR or by repeat professional Ag-RDT) COVID-19 diagnosed within three months, 6 clusters per arm of ~100 HCWs each, the study will have 84.1% power to detect the stated difference of harmonic count of 15.

Recruitment process

Recruitment at each site will be publicized by announcements at the morning clinical staff review meetings, and by asking the Nurse-in-Charge for time to meet with non-clinical staff for similar group talks. The project will be discussed, and leaflets developed by PSI-Malawi in collaboration with Ministry of Health will be distributed. The benefits of early diagnosis to prevent onward transmission to patients and family members, and for treatment (if eligible) once antivirals are available in Malawi, will also be discussed.

The study staff will establish tents on-site where participants will be consented. Participants will be asked in the meetings above to self-present at the study tents during lunch breaks and quiet clinical times. Individual written informed consent and baseline procedures will take place in these study tents. On attendance at the recruitment site, the research assistant will screen for eligibility and complete informed consent process before study enrolment. Any clinical questions will be referred to the project coordinator who is medically trained. Staff who present at the study site will be cross-referenced and checked off a list of all health care workers at each of the 12health facilities from the human resources department (using permission granted from the District Health Office).

Staff members who do not attend the tents within one week will be followed at their workstation by a study member to ask if they are intending to participate at all, answer questions and schedule a

meeting time at the study tent. For staff who do not wish to participate, they will be told that if they decline to participate in the study, they will not be punished, and such a decision will not be passed on to any of the health facility leadership. No further contact will be made with these staff. For staff who indicate interest in participating, but who have not yet self-presented to the study tent, up to 3 attempts will be made to recruit staff before non-participation is assumed. Additionally, for any health care workers who decline to participate but show COVID-19 symptoms will be encouraged to be tested through the existing government COVID-19 testing sites.

The staffing lists therefore will be used to cross-reference when participants self-identify for participation, knowing when all staff members are accounted for, as well as avoiding inconveniencing non-participating staff by contacting them more than once. These lists will also be used to generate an overall participation rate figure.

Inclusion criteria for healthcare workers
Healthcare workers shall be eligible to participate if they are:

1. Aged 18 years and above
2. All health workers employed by the above facilities, including nurses, doctors, clinic assistants, health surveillance assistants, clinical officers, community health workers and lay counsellors, administrators, and support staff such as receptionists and porters.
3. Willing and able to give written informed consent

Exclusion criteria for healthcare workers

A healthcare worker shall be ineligible to participate if they:

1. Have tested positive for COVID-19 and are still within the isolation period
2. Have any medical contraindication to anterior nasal swab (e.g., bleeding disorders, recent facial trauma)

Main analysis

The geometric mean count of number of HCWs who meet the primary outcome will be calculated, with a negative binomial model used to compute incidence rate ratios (IRR) and 95% confidence intervals (CI) comparing the intervention arm (PCI) with the standard of care arm (SOC). The analysis will also account for overdispersion. The number of health care workers per health facility will be used in the negative binomial model as an offset.

Ethical considerations for the health care worker model
Eligible participants will be given detailed information about the study and will be invited to participate (NC07). Written informed consent will be obtained before study procedures commences. Written informed consent also explicitly covers the potential for subsampling for the process evaluation explaining that those in the self-testing arm may be selected to be asked some additional questions about their experiences of the testing after 12 weeks which means the exit interview could take up to 2hours.

Written informed consent will also be obtained from the study providers for the 2 qualitative interviews (NC03).

5.1.2. Routine provider-delivered diagnostic COVID-19 Ag-RDT testing in out-patients

Objective

To evaluate the acceptability of professional use Ag RDT of COVID-19 Ag-RDT, proportions of patients testing who would have met eligibility criteria for oral antiviral therapy, and acceptability of use of KN95 masks for infection prevention for those who test positive in outpatients in Blantyre.

To compare costs and cost-effectiveness during implementation of the OPD model.

Outcome

The main outcome is the proportion OPD attendees screening positive for COVID-19 symptoms and accepting to be tested for COVID-19.

Secondary outcomes include an assessment of risk factors including vaccination history, socio-demographics and knowledge, attitudes, perceptions, and practices relating to COVID-19 transmission and prevention.

Overview

The 12 health facilities used for the health care worker use case will also be selected for implementation and evaluation of the OPD use case. The sites will be selected on the basis of having at least 100 health care workers and therefore high throughput for outpatients i.e., also serving more than 100 outpatients in any given day. Patients who test positive will be offered confirmatory testing and care under national guidelines. Training for use of existing and/or new Ag-RDTs will be provided to staff before implementation commences.

Routine provider-delivered diagnostic services using COVID-19 Ag-RDTs will be supported by PSI and District Health Office, Blantyre for patients presenting to clinics with symptoms of COVID-19 according to Malawi case-definitions and testing criteria. Services will be aimed at decentralising testing and meeting peak-testing demands during epidemic waves as part of facilitating early detection and enable effective infection prevention and control, including linkage to appropriate COVID-19 care.

We aim to offer COVID-19 testing to OPD attendees first by introducing the study while they wait for clinical consultation in a group and secondly one-on-one through a study introduced screen where sampling and testing will occur (Figure 2).

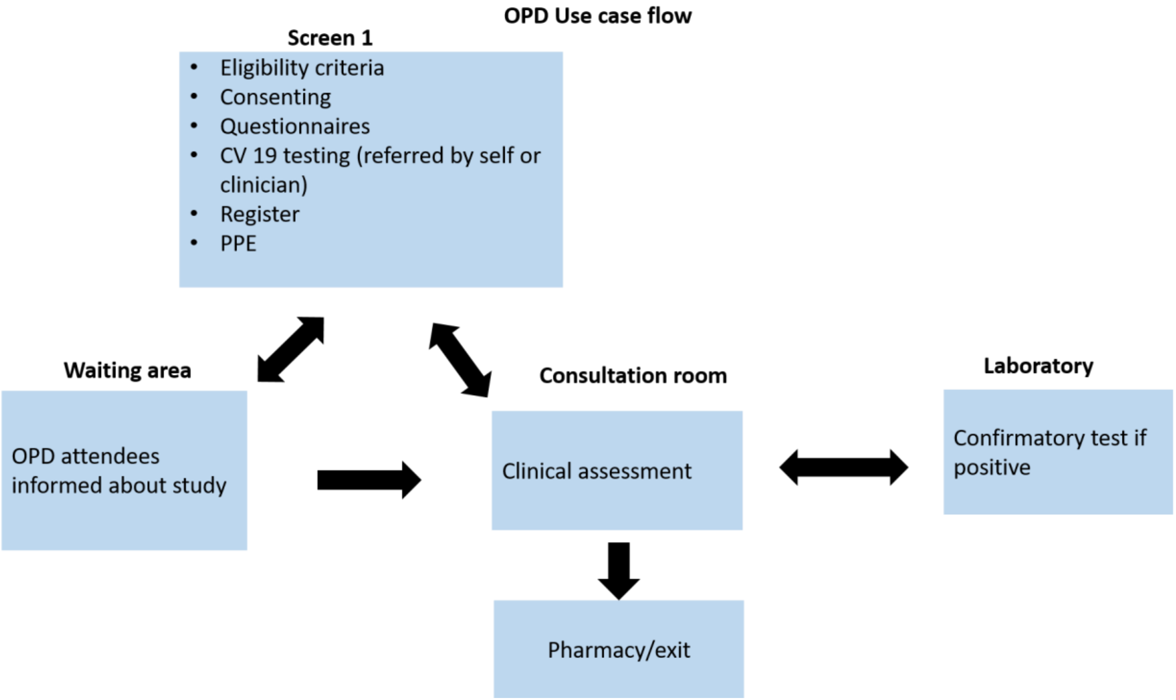

Figure 2: Patient flow at OPD

Any participant who tests positive with Ag-RDT may be asked for verbal consent to provide a confirmatory specimen for NAAT and viral sequencing in line with national policy in Malawi for patients who are not already part of an established outbreak and will be referred for clinical care in line with national guidelines.

High risk participants (risk factors for severe COVID-19, or severity of illness) will be triaged for safer home management initiatives and oral antivirals once these become available. All positive participants will also receive two KN95 face masks for themselves and a pack of surgical face masks for their household or other close contacts. The masks will be provided on demand to the case and their direct care providers. Participants testing positive will also be screened for eligibility for the COVID-19 contact-tracing study based on secondary-distribution of self-test kits described below.

Study activities

Epidemiological
A structured questionnaire (CP01) shall be used to collect data on the participants' socio-demographic characteristics, assess for COVID-19 symptoms, willingness to test and follow next steps on COVID-19 prevention if positive.

Qualitative
A subsample of 20 participants will be selected to ascertain the acceptability of the trial in line with Proctor's implementation outcomes (Client SSI).

Study coordinators and 2 study providers will be interviewed to write up the TIDieR description for this use-case, as well as to input into the acceptability of the use-case in line with Proctor's implementation outcomes (Provider SSI topic guide & CK01).

Cost and cost effectiveness

A subsample of 20 participants will be selected to receive a structured questionnaire asking clients about the time spent testing, and any costs incurred in accessing COVID-19 testing (Patient cost questionnaire_Final).

A maximum of 12 providers shall also be interviewed and observed through a time and motion approach, ascertaining the costs of overhead supplies (utilities, facility supervision, administrative, cleaning, security and transport costs), COVID-19 testing costs (Ag RDT or PCR), assessing rooms, equipment, furniture, staffing, consumables and equipment used for each COVID-19 testing service, and time and motion (method of collecting cost data) for staff providing COVID-19 testing services (Provider costing data collection tool).

Sample size

We conservatively assume that 70% of participants across clinics will accept to be tested for COVID-19. For the sample proportion to be estimated to within +/-0.05 (5%) using the 95% confidence level, a sample of 323 participants would be required.

However, we assume the following:

i. Integration of COVID-19 testing into routine health services will be maintained for the foreseeable future but will target people with symptoms of COVID-19 and for those who are at high risk for severe COVID-19.

ii. We also assume, based on precedent, that low proportions will test positive outside of an epidemic wave, with each epidemic wave lasting 6 weeks, and we expect 2 waves within the study period.

iii. We assume that each facility serves at least 100 OPD attendees per day, of whom at least 30% will have one or more COVID-19 symptoms, and that 70% of those eligible will accept SARS-CoV-2 testing i.e., 21 attendees could accept testing per day over 12 sites, totaling a potential study population of 252 in any one day, or 7,560 per 6week wave.

Recruitment process

Generally, the recruitment process for OPD attendees follows the flow described in Figure 1.

A research assistant placed at the recruiting health facility will be positioned at a temporary shelter mounted within the OPD space. As patients wait in a group, the research assistant will provide study information and offering patients the choice to participate in the study.

Those giving verbal consent will then be screened for eligibility and will complete an informed consent process one-on-one with the research assistant in the temporary shelter. Queue numbers will be provided so that the patient would maintain their position to ensure that the study doesn't unreasonably inconvenience the patient. Patients who refuse to participate in the study will continue with their medical consultation and study staff will not disclose such a decision to the clinician or medical staff.

Inclusion criteria

1. Aged 18 years and above

2. Willing and able to provide informed consent (written/thumbprint/verbal)

Exclusion criteria

1. Have tested positive for COVID-19 and are still within the isolation period
2. Have a medical contraindication to nasal swabs (e.g., bleeding disorders, recent facial trauma).
3. Are unable to provide informed consent

Main analysis

A programmatic evaluation and analysis of data will report on the proportion of participants accepting to be tested, tested positive and those linking to care and treatment. A descriptive process evaluation will analyze month-by-month the numbers of COVID-19 tests conducted at each facility before introducing COVID-19 Ag-RDTs. Once Ag-RDTs are made available, numbers accepting the test, and numbers diagnosed Ag-RDT+ve will be collected. Denominators will be all clinic attendees.

Time trend analysis will be used for descriptive analysis of trends in numbers (%) of all attendees screened for symptoms, reporting one or more COVID-19 symptom, identified, and tested for COVID-19, and the number/percentage of those testing positive. Data will be reported for all patients, and for all patients who would be eligible for antiviral treatment if tested positive.

Descriptive analysis of the proportion of participants and binomial exact 95% CI accepting to test, testing positive and accepting next steps for COVID-19. The proportions will be stratified and compared either using t-test or Chi-square test for continuous and categorical variables accordingly.

Ethical considerations for the OPD model use case
Eligible participants will be given detailed information about the study and will be invited to participate (NC08A). Written informed consent will be obtained before study procedures commences. Written informed consent also explicitly covers the potential for subsampling for the process evaluation explaining that clients may be selected to be asked some additional questions about their experiences of the testing which means that the interviews could take up to 1hour.

In addition, written informed consent will be obtained from participants subsampled to take part in the patient cost exercise (NC09), as well as written informed consent from the providers sampled to take part in the provider cost exercise (NC10). If participants are illiterate, participant information sheets will be read to participants and thumbprint consent, alongside witness signature will be taken.

5.1.3. Written informed consent will also be obtained from the study providers for the 2 qualitative interviews (NC03).Investigating potential COVID-19 outbreaks in congregate, populated settings

Objective
We wish to evaluate the acceptability of professional use Ag RDT and use of KN95 masks by the COVID-19 positive participant to prevent infection of contacts in congregate settings (universities, workplaces) within the catchment area of the PCI facilities in Blantyre **to expand peak-testing capacity** during outbreaks and to facilitate early detection and enable effective infection prevention and control, including linkage to appropriate care.

Outcome

The primary outcome is the proportion accepting to be tested for COVID-19.

Secondary outcomes include an assessment of risk factors including vaccination history, socio-demographics and knowledge, attitudes, perceptions, and practices relating to COVID-19 transmission and prevention.

Overview

An outbreak is defined as the occurrence of ≥1 new confirmed case in any congregate setting. We will actively work with district-wide COVID-19 response teams to get weekly reports of any such new cases.

Criteria for selecting a congregate setting for implementation of screening:

1. A working relationship must exist between the potential congregate setting and the district response team.
2. Must be one of the two below:
    a. a university
    b. a workplace
3. A gatekeeper must provide consent at institutional level for the implementation of screening.

We will select at most 5 congregate settings aiming to offer screening to ~323 participants (i.e. aiming to recruit a total of 323 participants across all 5 settings). Assuming that there would be at most two waves during study implementation period then two outreach screening activities would be conducted.

Professional Ag-RDT will be used with national guidelines followed for confirmation as well as linkage to care.

Suspected outbreaks of COVID-19 will be investigated among individuals in heavily populated environments in Blantyre such as universities and workplaces to facilitate early detection and enable effective infection prevention and control, including linkage to appropriate COVID-19 care. We will conduct programmatic evaluation of routine data collected by PSI and implementing partners to provide analysis of time trends and possible contribution of COVID-19 Ag-RDT provider-delivered testing. PSI will implement the testing and facilitate linkage to care and treatment and will collect programmatic data.

Activities
Epidemiological
A structured questionnaire (CP01) shall be used to collect data on the participants' socio-demographic characteristics, assess for COVID-19 symptoms, willingness to test and follow next steps on COVID-19 prevention if positive.

Qualitative
A subsample of 20 participants will be selected, 5 per outreach site, to ascertain the acceptability of the use-casein line with Proctor's implementation outcomes (Client SSI).

Study coordinators and 2 study providers will be interviewed to write up the TIDieR description for this use-case, as well as to input into the acceptability of the use-case in line with Proctor's implementation outcomes (Provider SSI topic guide & CK01).

Cost and cost effectiveness

A subsample of 20 participants will be selected to receive a structured questionnaire asking clients about the time spent testing, and any costs incurred in accessing COVID-19 testing (Patient cost questionnaire_Final).

A maximum of 12 providers shall also be interviewed and observed through a time and motion approach, ascertaining the costs of overhead supplies (utilities, facility supervision, administrative, cleaning, security and transport costs), COVID-19 testing costs (Ag RDT or PCR), assessing rooms, equipment, furniture, staffing, consumables and equipment used for each COVID-19 testing service, and time and motion (method of collecting cost data) for staff providing COVID-19 testing services (Provider costing data collection tool).

Sample size considerations

We conservatively assume that 70% of participants the targeted outreach settings will accept to be tested for COVID-19. For the sample proportion to be estimated to within +/-0.05 (5%) using the 95% confidence level, a sample of 323 participants would be required.

In all congregate settings we will aim to recruit for research every 2nd eligible person aiming to stop when the overall sample size of 323 is reached.

Recruitment process

Below are the steps to be followed before, during and after screening for eligibility in congregate settings by the study team. Upon notification of a COVID-19 case at any of the selected congregate settings either via PSI or directly or via any other media, the project coordinator will contact the leadership of the institution.

Once confirmed and authorized the study team will then visit the institution within at most a day after being given a go ahead by the leadership of the institution. All available occupants of the congregate institution present at the time will then be screened for eligibility.

Before commencing any data collection, all eligible individuals at the institution will be provided information sheets to read followed by informed consent process completed by a research assistant.

Potential participants who may require more time will be allowed to read information sheets with potential participation on the next day following consent. The project coordinator who is medically trained will be available onsite to answer any clinical questions. Any prospective participant who declines to participate in the study will not be punished and such a decision will not be passed onto any of the institution. Additionally, for prospective participants who decline to participate but show COVID-19 symptoms will be encouraged to be tested through the existing government COVID-19 testing sites.

Inclusion criteria for people in populated settings

Residents of heavily populated environments shall be eligible to participate if they are:

1. Aged 18 years and above

2. Students/employees or residents of the institution or community where an outbreak is being investigated
3. Well enough to complete study activities
4. Willing and able to provide informed consent (written/thumbprint/verbal)

Exclusion criteria for people in populated settings

Residents of heavily populated environments shall be ineligible to participate if they:

1. Have tested positive for COVID-19 and are still within the isolation period
2. Have a medical contraindication to nasal swabs (e.g. bleeding disorders, recent facial trauma).
3. Are unable to provide informed consent

Main analysis

Descriptive analysis of the proportion of participants and binomial exact 95% CI accepting to test, testing positive and accepting next steps for COVID-19. The proportions will be stratified and compared either using t-test or Chi-square test for continuous and categorical variables accordingly.

Ethical considerations for outbreak investigation use case

Eligible participants will be given detailed information about the study and will be invited to participate (NC08B). Written informed consent will be obtained before study procedures commences. Written informed consent also explicitly covers the potential for subsampling for the process evaluation explaining that clients may be selected to be asked some additional questions about their experiences of the testing which means that the interviews could take up to 1hour.

In addition, written informed consent will be obtained from participants subsampled to take part in the patient cost exercise (NC09), as well as written informed consent from the providers sampled to take part in the provider cost exercise (NC10). If participants are illiterate, participant information sheets will be read to participants and thumbprint consent, alongside witness signature will be taken.

Written informed consent will also be obtained from the study providers for the 2 qualitative interviews (NC03).

5.1.4. Secondary distribution of self-test kits

Objectives:

To evaluate the acceptability of unsupervised self-testing provided through secondary distribution of COVID-19 Ag-RDT self-test kits to contacts of patients testing positive for COVID-19.

Outcome:

Proportion of contacts accepting the offer of COVID-19 self-testing from the index client.

Secondary outcomes include an assessment of risk factors of the contacts including vaccination history, and socio-demographics.

Overview

There will be a secondary distribution of self-test kits demonstration project to facilitate early detection of positive cases and appropriate linkage to COVID-19 care (home or facility based).

COVID-19 positive patients (index cases) from the HCW, OPD and Outbreak investigation in congregate setting studies will be screened for eligibility and asked for informed consent to participate. The national guidance is that all close contacts of an index case should be deemed positive and treated as such (32). However, the decision to abandon contact tracing in Malawi was largely taken due to the labour and resource-intensive nature of professional Ag-RDTs or PCR for contacts.

This study's approach is to offer COVID-19 self-test kits to the contacts and collect data via the index case, which is relatively inexpensive. Making a generalized assumption that all contacts should be deemed positive may inadvertently put individuals at the front of stigma and economic exclusion.

There are very limited data on COVID-19 secondary distribution in low-and-middle-income-countries and this is a good opportunity to test the model which may as well become the mainstay to manage large testing volumes in times of great demand during COVID-19 waves.

Participants will be provided with brief training on how to demonstrate correct use of nasal swabbing and testing and information leaflets including direction on how to access web-based or WhatsApp video clips.

Activities

Epidemiological

A structured questionnaire (CC01) shall be used to interview index cases who tested positive for COVID-19 from any of the use cases at time of testing positive. The questionnaire will collect information on social demographic indicators about the index, as well as COVID-19 symptoms, risk and testing, and information about their close contacts. The index will themselves fill in data on their contacts onto a contact log (CC03).

A second structured questionnaire (CC02) will be used to interview index cases 7-14 days after recruitment to follow up on the details of each of their listed contacts via telephone interview (the index will use their log to report this data back). Please note at no point do we call the contacts themselves.

Qualitative
A subsample of 20 index patients will be selected to ascertain the acceptability of the use-case in line with Proctor's implementation outcomes (Client SSI).

Study coordinators and 2 study providers will be interviewed to write up the TIDieR description for this use-case, as well as to input into the acceptability of the use-case in line with Proctor's implementation outcomes (Provider SSI topic guide & CK01).

Cost and cost effectiveness

A subsample of 20 participants will be selected to receive a structured questionnaire asking clients about their time spent testing, and any costs incurred in accessing COVID-19 testing (Patient cost questionnaire_Final **Secondary**). This tool is different to Patient cost questionnaire_Final tool used in the other use-cases, as it seeks to understand costs of delivering the test kits to contacts, instead of the costs of receiving a test. Written consent is taken in advance, and the interview is administered on day 14 during the follow up phone call.

Sample size

The number of contacts will be determined by the number of index cases and is not known at the time of design. However, assuming 2% of all our screening tests from the above use cases are positive i.e., ~30,000 overall tests, then about 600 index cases will be generated. Assuming there would be three household contacts and 2 two close contacts, then a total of 3000 contacts will be approached. When oral antivirals become available in Malawi, information on treatment eligibility and how to access antivirals will be included.

For statistical power with the secondary distribution of self-test kits, we assume 60% of contacts will be willing to self-test for COVID-19 if offered. For the sample proportion to be estimated to within +/-0.05 (5%) using the 95% confidence level, a sample of 370 contacts accepting self-tests is required.

Recruitment process

Recruitment process for secondary distribution of self-test kits will begin with a positive COVID-19 test (index). The same research assistant who conducted the initial consenting and baseline data collection for the other use cases will screen the index case for eligibility and complete the written informed consent for the index. All consenting index cases will then be enrolled in the study. Index cases who decline to participate in secondary distribution of self-test kits component of the study will not be disadvantaged in any way and can opt out of the study if no other follow-up activities are due.

Following written, informed consent, index clients will be shown the self-test kits and how to use them and asked to list the fully anonymized details of any household or other close contacts aged 18 years or above. The index will maintain their own contact list using a Paper Enrollment Log (CC03) to assist them with their organization, distribution and documentation of any subsequent steps taken after testing and reporting back to us. If indicated at this stage, written informed consent will also be taken for the costing piece.

At 14 days after recruitment, the data from the enrollment log will be reported back by the index during a telephone interview. The study team will enter this data into simple aggregate format that will be entered electronically as a single aggregate record for each index case (number of contacts; numbers aged over 65 years; numbers offered self-testing; numbers accepting Day 1 self-testing etc.) provided with a) two KN95 masks for themselves; b) a pack of surgical masks to offer to their contacts; and c) two self-test kits to give to each named contact for use immediately and on day 5.

The proportion of contacts linked to care will also be measured and reported. All contacts will be provided with a study leaflet (NC06B) via the index client that explains clearly where to seek care if the self-test result is positive. Additionally, the leaflet provides phone numbers for the project staff who will be on hand to direct positive contacts to the nearest clinic that provides COVID-19 care.

Outcomes will be collected by telephone interview with the index case two weeks after enrolment. The data from the phone interview will include if the index delivered the COVID-19 self-test kits to the contacts or not, contact used the test kits as advised and if they disclosed the results to index. No personal identifiers will be collected on the contacts, but we will ask the index to identify any known high-risk factors (age over 65 years, or over 50 years with co-morbidity) that would be a potential indication of eligibility for outpatient treatment of the tested contact. If consent has been taken, the participant cost survey will then be administered over the phone as well.

A person shall be considered a contact if they had any one of the following exposures to a confirmed case as suggested by WHO(33):

- face-to-face contact with a confirmed case within 1 meter and for at least 15 minutes;
- direct physical contact with a confirmed case;
- direct care for a patient with probable or confirmed COVID-19 disease without the use of recommended PPE.

Inclusion criteria

Index case patients shall be eligible for inclusion if they are:

1. Tested positive for COVID-19
2. Have at least one adult close contact
3. Do not feel at risk of physical harm, loss of employment or loss of housing from participating
4. Feeling well enough to comfortably conduct the study activities, as determined by participant reports and assessment by study staff.

Exclusion Criteria for index case patients

Index case patients shall be ineligible for participation if they are

1. Are unable or unwilling to provide informed consent
2. Not traceable or able to be interviewed by telephone
3. Unable to demonstrate back how to self-test correctly

Main analysis

The main analysis relates to exploratory analysis of acceptability (% eligible who accept the offer of secondary distribution of kits to household or other close contacts) and contact acceptability (% contacts who self-test), safety (self-report of any social harms experience by the index patient), numbers of kits distributed, numbers of contacts accepting kits, number of contacts testing at least once, number of contacts with positive immediate or day 5 results, and number of contacts diagnosed within 5 days of symptom onset including asymptomatic contacts.

Qualitative data analysis will be captured by semi-structured interview during which index patients will also be asked about any perceived benefits and regrets, and their willingness to recommend secondary distribution to others testing positive for COVID-19.

Ethical considerations for the secondary distribution model

Eligible index participants will be given detailed information about the study and will be invited to participate (NC06A). Written informed consent will be obtained before study procedures commences. Written informed consent also explicitly covers the potential for subsampling for the

process evaluation explaining that clients may be selected to be asked some additional questions about their experiences of the testing which means that the interviews could take up to 1hour.

Study information flyers will be distributed by the index patient to their contacts as part of the consent waiver. Consent requirements for each research activity.) Social harms will be expressly solicited via telephone call. No under 18s will be recruited for secondary contacts.

In addition, written informed consent will be obtained from index participants subsampled to take part in the patient cost exercise (NC09) at the point of diagnosis. If participants are illiterate, participant information sheets will be read to participants and thumbprint consent, alongside witness signature will be taken.

Written informed consent will also be obtained from the study providers for the 2 qualitative interviews (NC03).

5.2. Study place

12 health facilities shown below will be allocated for the non-randomized study:

	**Programmatic COVID-19 Intervention (PCI) arm**	**Standard Of Care (SOC) arm**
**Facility**	Bangwe Health Center	Chilomoni Health Center
	Chileka Health Center	Mpemba Health Center
	Dziwe Health Center	Lirangwe Health Center
	Mdeka Health Center	South Lunzu Health Center
	Chichiri Health Center	Limbe Health Center
	Ndirande Health Center	Zingwangwa Health Center

The 12 health facilities in Blantyre used for the health care worker use case above will also be selected for implementation and evaluation of the OPD use case.

Criteria for selecting a congregate setting in Blantyre for implementation of screening will be:

4. A working relationship must exist between the potential congregate setting and the district response team.
5. Must be one of the two below:
    a. a university
    b. a workplace
6. A gatekeeper must provide consent at institutional level for the implementation of screening.

5.3. Study population

All HCWs (including administrators, maintenance staff, support staff, and clinicians) working in the targeted 12 health facilities of Blantyre District will be offered the opportunity to participate in the non-randomized controlled study.

Inclusion criteria for healthcare workers

Healthcare workers shall be eligible to participate if they are:

4. Aged 18 years and above
5. All health workers employed by the above facilities, including nurses, doctors, clinic assistants, health surveillance assistants, clinical officers, community health workers and lay counsellors, administrators and support staff with direct patient contact such as receptionists and porters.
6. Willing and able to give written informed consent

Exclusion criteria for healthcare workers

A healthcare worker shall be ineligible to participate if they:

3. Have tested positive for COVID-19 and are still within the isolation period
4. Have any medical contraindication to anterior nasal swab (e.g. bleeding disorders, recent facial trauma)

Inclusion criteria for people in populated settings

Residents of heavily populated environments shall be eligible to participate if they are:

5. Aged 18 years and above
6. Students/employees or residents of the institution or community where an outbreak is being investigated
7. Well enough to complete study activities
8. Willing and able to provide informed consent (written/thumbprint/verbal)

Exclusion criteria for people in populated settings

Residents of heavily populated environments shall be ineligible to participate if they:

4. Have tested positive for COVID-19 and are still within the isolation period
5. Have a medical contraindication to nasal swabs (e.g. bleeding disorders, recent facial trauma).
6. Are unable to provide informed consent

Inclusion criteria for Outpatient Department attendees

OPD attendees will be eligible to participate if they are:

1. Willing and able to provide written informed consent
2. Feeling well enough to comfortably conduct the study activities, as determined by participant reports and assessment by study staff.

Exclusion criteria for Outpatient Department attendees

OPD attendees will be ineligible to participate if they:

1. Have tested positive for COVID-19 and are still within the isolation period
2. Have any medical contraindication to anterior nasal swab (e.g. bleeding disorders, recent facial trauma)
3. Are unable to provide informed consent

For contact tracing component:

Inclusion criteria for index case patients

Index case patients shall be eligible for inclusion if they are:

5. Tested positive for COVID-19
6. Have at least one adult close contact at their place of residence
7. Do not feel at risk of physical harm, loss of employment or loss of housing from participating
8. Feeling well enough to comfortably conduct the study activities, as determined by participant reports and assessment by study staff.

Exclusion Criteria for index case patients

Index case patients shall be ineligible for participation if they are

1. Are unable or unwilling to provide informed consent
2. Not traceable or able to be interviewed by telephone
1.

Exclusion criteria for contacts

Contacts shall be ineligible for participation if they are

1. Are unable to provide informed consent
2. Have any medical contraindication to anterior nasal swab (e.g. bleeding disorders, recent facial trauma)

For the costing component we will further conduct exit interviews on 100 purposively selected testers across all use cases.

5.4. Qualitative process evaluation

This data collection activity is an additional activity within each use-case to generate evidence on how best to optimize the delivery of the interventions at scale. A TIDieR (Template for Intervention Description and Replication) summary for each use case will be written up, ensuring that each use-case is reported to the minimum standard to ensure replicability, of interventions. To create the TIDieR framework, semi-structured interviews with the study coordinators will be conducted, complemented by interviews with two implementers on the ground for each use-case (CK01).

Thereafter, the study shall use Proctor's framework (Table 2) for implementation outcomes to evaluate each use-case according to the acceptability, appropriateness, feasibility, and fidelity domains below:

Table 2. Proctors' implementation outcomes

Implementation outcome	Level of analysis	Available measurement
Acceptability	Participants across all use cases<br>+ 2 implementers per use case	Semi-structured interviews
Adoption (uptake)	PSI programme	Programme data for each use case:<br>• Number tested<br>• Number linked to care<br>• Number receiving care
Appropriateness	Participants across all use cases<br>+ 2 implementers per use case	Semi-structured interviews
Costs	Costing data across all use cases	Costing and cost effectiveness study<br>Health system, user costs and societal downstream costs.
Feasibility	Participants across all use cases<br>+ 2 implementers per use case	Semi-structured interviews
Fidelity	Participants across all use cases<br>+ 2 implementers per use case	Semi-structured interviews
Penetration	PSI programme	Scaled to other programme sites

The participant sampling strategy for each semi-structured interview will follow the framework in Table 3 below:

Table 3. Sampling strategy for use-cases semi structured interviews:

**Use case and participant group**	**Sampling strategy**	**Estimated number of semi-structured interviews**
1. Health care workers	10 participants from self-testing and 10 participants from diagnostic testing facilities	Estimate 20 people (saturation)
2: Outbreak	5 recruited per outreach site from 4 different locations	Estimate 20 people (saturation)
3: Primary care facility OPD	Recruit 10 participants from 2 sites	Estimate 20 people (saturation)
4: Secondary distribution of self-test kits	Interview index patients (those that agree to linkage/ follow up).	Estimate 20 people (saturation)
**Totals**		**80 interviews**

5.5. Costing and cost-effectiveness

The one-off testing models of COVID-19 testing, and self-testing will be fully costed to provide cost data that may be anticipated under programmatic implementation for policy makers. Therefore, this cross-cutting component aims to collect cost data that will be essential to cost and cost effectiveness analyses, to be used in the cost section of the process evaluation.

Objectives:

1. To conduct costing of the implementation models
2. To compare costs and cost-effectiveness between period before and during implementation for the OPD model
3. To undertake economic modelling to estimate the impact of the different models.

Methodological approaches

We will cost the OPD, outbreak and secondary distribution use cases using the iDSI Reference Case for economic evaluation guidelines (i.e., one-off testing models). Health care worker regular self-testing is explicitly **excluded**. The costing will be conducted from a societal perspective using ingredients-based costing approach. We will combine top-down and bottom-up costing approaches:

The bottom-up approach will include a time and motion costing approach aimed at observing staff time allocation on a typical workday, (12 providers in OPD and outbreak investigations), given there are no staff time equivalents with the secondary distribution model, totalling 24 providers.

Time and motion refer to providers working on multiple activities across all use cases. For example, a provider conducting both self-testing and PCR in a healthcare facility. Such a provider will be observed using time and motion studies to determine allocation of personnel time to professional use testing and PCR. The time and motion costing approach is not a new study in its own right, but a method of cost data collection as part of the cost and cost effectiveness component of the project. Written consent will be obtained ahead of the time and motion observations.

We will further conduct exit interviews on 60 purposively selected testers across the OPD, outbreak and secondary distribution use-case. These will be done at exit of the process and will be aimed at collecting costs of accessing COVID-19 testing. We will ask participants to estimate the amount of time taken and costs incurred to access testing. This will enable the estimation of opportunity cost to test both as direct out-of-pocket payments (direct costs) and lost income (indirect costs).

Since we are only interested in costs of accessing testing, we will not include any additional downstream costs including household costs. Written informed consent will be obtained from all participants before the interviews.

Recruitment and consenting process

Participant interviews: The project coordinator will liaise with the recruitment site leadership to identify participants for all costing components. All those who consent to participate will be interviewed on site by a research assistant after their testing is completed. A small subsample will also be observed accessing the services from arrival at the centre to understand the patient pathway. We aim to recruit twenty participants for interviews per one-off testing use case. The secondary distribution of test-kit participants will be consented with written informed consent

before they leave the clinic, and the interview will take place on day 14. Recruitment and consenting for participants identified during implementation of study interventions will also be completed by the research assistant soon after receiving the testing at the recruiting site.

Provider interviews and observations: For use cases where testing will be happening even on weekends, the sample will be split to 10 observations on a weekday and 10 observations on a weekend. Half of the observations will be in the morning and the other half in the afternoon to ensure we are observing different type of clients.

All our costs and the associated ingredients and allocation factors will be shared with mathematical modellers from the London School of Hygiene and Tropical Medicine (LSHTM) and the Health Economics and Epidemiology Research Consortium for additional economic modelling.

Outcomes

i. Cost per kit distributed
ii. Cost per kit used
iii. Cost per positive patient diagnosed
iv. Cost per positive patient diagnosed and linked to prevention and care
v. Cost per COVID-19 infection and death averted
vi. Cost of scaling-up COVID-19 self-testing

5.6. Clinical aspects and PPE

Any of the use cases who test positive for COVID-19 will be notified of their results and managed according to disease severity as per available local guidelines.(34) Asymptomatic cases will be advised to self-isolate, monitor for COVID-19 symptoms, and report to the nearest health facility if symptoms emerge or worsen. COVID-19 positive cases with symptoms will be assessed for risk factors and severity (mild, moderate or severe) and referred to collaborating health facility for supportive treatment as required. All positive cases shall receive two KN95 masks for themselves and a pack of surgical masks to offer to their contacts. They shall also be advised on COVID-19 prevention measures including the proper use of face masks, social distancing, hand hygiene, and cough and sneeze etiquette.

5.7. Study period

Figure 3: project timelines

	2022			
**Activity**	Q1	Q2	Q3	Q4
Protocol and tools development				
Ethics Approvals				
Staff hiring & training				
a. Facility-based Ag-RDT CV19 interventions				
i. Intensified diagnosis and screening in HCWs				
1. Diagnostic HCW clinic, risk-based home-management				
2. Twice weekly self-testing for early diagnosis				
ii. Increased capacity and efficiency in OPD				
1. Systematic screening for CV19 symptoms + diagnostic testing				
2. Add self-testing to improve surge management/efficiency				
3. Secondary distribution of self-test kits: contacts				
b. Early diagnosis in communities				
i. Outreach testing: suspected congregate outbreaks				
1. Provider-delivered Ag-RDT testing				
2. Add community-led self-testing to increase timeliness/sustainability				
c. Safer and more affordable cross-border travel (all provider-delivered)				
i. Evaluate Ag-RDT vs NAAT in returning travelers				
ii. Add Ag-RDT services/masks to land borders				
Economics				
Dissemination				
Publications				

5.8. Overall sample size considerations

For the non-randomized controlled study: with an assumed harmonic mean number of 20 and 35 HCWs (common standard deviation: 12) in SOC and the PCI arm confirmed (either by PCR or by repeat professional Ag-RDT) COVID-19 diagnosed within three months, 6 clusters per arm of ~100 HCWs each, the study will have 84.1% power to detect the stated difference of harmonic count of 15.

For components offering self-testing, we conservatively assume that 60% of participants will be willing to self-test for COVID-19 if offered. For the sample proportion to be estimated to within +/-0.05 (5%) using the 95% confidence level, a sample of 369 participants would be required. Whereas for components offering professional COVID-19 Ag-RDTs we conservatively assume that 70% of participants in each of the targeted groups will accept to be tested for COVID-19. For the sample proportion to be estimated to within +/-0.05 (5%) using the 95% confidence level, a sample of 323 participants would be required.

5.9. Data collection

Process evaluation

A TIDieR (Template for Intervention Description and Replication) summary for each use case will be written up, ensuring that each use-case is reported to the minimum standard to ensure replicability, of interventions. To create the TIDieR framework, semi-structured interviews with the study

coordinators will be conducted, complemented by interviews with two implementers on the ground for each use-case.

Thereafter, the study shall use Proctor's framework for implementation outcomes to evaluate each use-case.

For the nonrandomized controlled study:

Brief baseline data will be collected at the time of written informed consent to participate in each of the 12 facilities. The primary outcomes will be based on incident diagnosis of COVID-19, with the aim being to increase early diagnosis in the self-testing facilities compared to a) pre-intervention rates through retrospective data extraction from HCW data from the facilities and prospective interviews with HCWs and b) between the two different testing strategies. Additional outcomes will be captured at exit interview, during which HCWs will be asked about any time off work, and reason for this (any positive COVID-19 tests or untested 'flu like illnesses and any quarantine due to close contact). An assessment of risk factors including vaccination history, and knowledge, attitudes, perceptions and practices relating to COVID-19 transmission and prevention will also be explored and compared by facility testing-strategies. Cost effectiveness analysis will be conducted as detailed in the economics section for this component of the project.

Overall data tools

Data tools for the different components of the project have been developed by the investigators. For the HCW component, potential participants shall be screened for eligibility (Appendix 7 - CH00: Eligibility screening) before recruitment. A structured questionnaire (Appendix 8 - CH01A :Healthcare workers baseline questionnaire) shall be used to collect data on the participants' demographic characteristics, assess their risk for severe COVID-19, knowledge on basic facts about COVID-19 and their practices and behaviours regarding COVID-19 prevention at baseline in both study arms. Another structured questionnaire (Appendix 9- CH02: Healthcare worker follow-up) shall be used to follow-up the participants monthly up to three months to find out if they had any COVID-19 symptoms over the past four weeks. For those who had the symptoms, we will ask about the management and care they received. After the three months follow-up period, a structured questionnaire (Appendix 10 - CH03: Healthcare worker exit) shall be used to conduct an exit interview where we will reassess the participant's risk for severe COVID-19, knowledge on basic facts about COVID-19, absenteeism from work due to COVID-19, their practices and behaviours regarding COVID-19 prevention, COVID-19 related stigma and discrimination, and experiences with COVID-19 self-testing.

For the PSI-led provider-delivered COVID-19 Ag-RDT testing at OPDs and congregate settings, eligibility screening shall be conducted using Appendix 11 (CP00) and Appendix 13 (CW00) respectively. Structured questionnaires (Appendix 12- CP01: OPD Questionnaire and Appendix 14 – CW01) shall be used to collect data on the participants' socio-demographic characteristics, assess for COVID-19 symptoms, willingness to test and follow next steps on COVID-19 prevention if positive.

For the contact tracing study, potential participant shall be screened for eligibility (Appendix 15 - CC00: Eligibility screening) before recruitment. A structured questionnaire (Appendix 16 - CC01:

baseline questionnaire for index case) shall be used to interview index cases who tested positive for COVID-19 from any of the use cases at time of testing positive. The questionnaire will collect information on social demographic indicators about the index, COVID-19 symptoms, risk and testing, and information about their close contacts. Another structured questionnaire (Appendix 17- CC02: Index case follow up questionnaire) will be used to interview index cases 7-14 days after recruitment to follow up on each of their listed contacts.

For the qualitative process evaluation study, semi-structured topic guides shall be used to interview clients from OPD, congregate settings (Appendix 18: Client process evaluation qualitative interview guide), implementers (study coordinators, investigators and frontline implementers) (Appendix 19 - CK01: Process evaluation among key informants) and HCW (Appendix 20 - Providers SSI topic guide) in PIC arm who would have completed the three months follow-up period to assess the acceptability, adoption, appropriateness, costs, feasibility, fidelity and penetration of the intervention.

For the cost and cost effectiveness study, a structured questionnaire (Appendix 22 - Facility costing data collection tool) shall be used to interview clinic managers on: costs of overhead supplies (utilities, facility supervision, administrative, cleaning, security and transport costs); and COVID-19 testing costs (Ag RDT or PCR). The tool shall also be used to assess rooms, equipment, furniture, staffing, consumables and equipment used for each COVID-19 testing service, and time and motion for staff providing CIVID-19 testing services. A semi-structured interview guide shall be used to ask clients about the time spent at the clinic and any costs incurred in accessing COVID-19 testing.

For the costing component: We will conduct exit interviews (Appendix 23: Client cost exit questionnaire) on 100 purposively selected testers across all use cases. The exit interviews will be aimed at collecting costs of accessing COVID-19 testing. We will ask participants to estimate the amount of time taken and costs incurred to access testing. For the OPD model, we will collect the user costs before and after the intervention to allow for a before and after analysis. This will enable the estimation of opportunity cost to testing both as direct out-of-pocket payments (direct costs) and lost income (indirect costs).

5.10. Data management and analysis

5.10.1. Data management

Data will be managed through infrastructure set up within Malawi-Liverpool-Wellcome Trust Clinical Research Programme (MLW). Data collection and processing will be as detailed in the data management plan (DMP) that will be developed for the project. Data will be collected using tablets running Open Data Kit (ODK) in Chichewa and will be downloaded onto a server running a MySQL Relational Database. Data and paper-based records of this project will be destroyed after 10 years. The data collected in this project will be anonymized by removing all information that may lead to identification of any of the study participants before being shared publicly through the London School of Hygiene & Tropical Medicine online repository (https://datacompass.lshtm.ac.uk). The full anonymization process will occur following peer reviewed publication but all contact details collected from participants through the study will be destroyed at the end of study follow-up for

data cleaning and query resolution. The College of Medicine Research Ethics Committee (COMREC) will monitor the study and thus will have access to participant information.

Data quality assurance will be implemented within the electronic form so that out-of-range values, inconsistent values and required variables will be checked at the time of data collection. All tablets will have full log-in details of the person collecting the data including a password. Access to the study database will be protected by a password known only to the PI (Augustine Choko) and the IT systems administrator in MLW. Data for study monitoring will be periodically exported into comma separated values (CSV) from the study database on the MLW server for analysis and to raise plus resolve data queries.

Protocols for managing data without breach of confidentiality are in place within MLW. Access to the final data set will be limited to the PI (Augustine Choko), co-principal investigators and colleagues listed as co-investigators.

Participants will be identified only by a unique study identifier on the data capture tools and any electronic database, with the exception of written screening and recruitment logs. All documents will be stored securely and only accessible by trial staff and authorised personnel. The trial will comply with the Data Protection Act, which requires data to be anonymised as soon as it is practical to do so.

All devices and paper-based tools containing data will be kept in locked offices at MLW during data processing and in a locked data repository room for longer term storage. All data will be backed up daily by the MLW Data Office, with offsite back up once weekly. Backup data will be stored in a locked filing cabinet away from the office by the PI for up to 10 years after study completion. No samples collected from this study will be stored after testing.

Dr Nicola Desmond from Liverpool School of Tropical Medicine and Dr Wezzi Lora from the MLW are leading the qualitative aspects of the project and are extremely experienced social scientists. The qualitative data will be managed using NVIVO software which is already available with an institutional license at MLW and is currently already and installed and in use by various projects for Dr Desmond.

5.10.2. Statistical analysis

Analyses will use R(35) and Stata 14.0 (Stata Corp, Texas, USA). Baseline characteristics will be computed as proportions or median (interquartile range [IQR]) as appropriate. Estimates such as proportions and mean will be reported along with 95% confidence intervals (CI). Regression analysis: logistic for binary outcomes and linear for the turn-around-time or any other continuous outcome will be explored.

5.10.3. Qualitative data analysis

All interview recordings will be transcribed and where appropriate translated into English. For the stakeholder interviews we will follow a deductive analysis approach using framework analysis following the domains identified within the TIDieR checklist for stakeholders across each site.

Emergent themes within the deductive framework will be identified through an inductive coding approach to identify the key components of each of the thirteen checklist items. Data will be presented comparatively for each component intervention. For process evaluation interviews we will follow the Proctor taxonomy of implementation outcomes comparing across different groups of participants for each component intervention. This will follow an inductive approach for emergent themes aligned within each implementation outcome category through a process of constant comparison across participants, triangulating different perspectives. Since implementation outcomes are often interrelated and dynamic we will examine the interrelatedness of themes as they emerge across different categories and whether these change over time or across sub-groups. The findings will help to inform our interpretation of intervention outcomes.

5.11. Results presentation

Numerical data will be presented graphically using appropriate techniques following data analysis. For example comparison of geometric mean count of numbers with COVID-19 by health facility and study arm will be displayed using a chart in R statistical software. The majority of the results will be presented in tabular form.

The major themes emerging from the qualitative data will be presented in textual form with comparisons by sex and age among *a priori* factors.

5.12. Dissemination of results

Study findings will be primarily aimed to inform the Ministry of Health (MoH) through presentations and final copy of the report. Further local dissemination will be done at the KUHES annual dissemination conference. Findings will also be presented at peer-reviewed regional and international conferences. Copies of the final report; published peer-reviewed paper (s) and abstracts will be made available to the KUHES Library, and to COMREC.

6. Overall ethical considerations

6.1. Ethics approval

Ethics approval will be sought locally from the College of Medicine Research and Ethics Committee (COMREC) in Blantyre, Malawi and internationally from the World Health Organization Ethics Review Committee in Geneva, Switzerland and the London School of Hygiene & Tropical Medicine Ethics Committee. Written or thumb print informed consent will be taken from all participants except for services deemed to be standard of care under the national guidelines such as confirmation of a positive COVID-19 RDT test and treatments.

The principles of GCP will be adhered to for the research components, and all research staff will have certified GCP training and will adhere to ICH-GCP requirements for source documentation and storage.

Eligible index participants will be given detailed information about the study and will be invited to participate (NC06A). Written informed consent will be obtained before study procedures commences. Written informed consent also explicitly covers the potential for subsampling for the

process evaluation explaining that clients may be selected to be asked some additional questions about their experiences of the testing which means that the interviews could take up to 1hour.

Study information flyers will be distributed by the index patient to their contacts as part of the consent waiver. Social harms will be expressly solicited via telephone call. No under 18s will be recruited for secondary contacts. More information on this is included in section 7 anticipated benefits and harms.

In addition, written informed consent will be obtained from index participants subsampled to take part in the patient cost exercise (NC09) at the point of diagnosis. If participants are illiterate, participant information sheets will be read to participants and thumbprint consent, alongside witness signature will be taken.

Written informed consent will also be obtained from the study providers for the 2 qualitative interviews (NC03).

6.2. Written or witnessed informed consent

Written or witnessed informed consent (thumb print) will be taken from all participants included in the project except for the secondary distribution use-case where we request **waiver of informed consent** for anonymised data collection of contacts, by the index patient.

The request for waiver of consent to test and to collect data assumes very low (close to minimal) individual risks of this approach, with 100s of millions of self-testing episodes already carried out for COVID-19 globally, combined with the benefits of easy access to timely testing for those same individuals, in addition to the extremely high public health value of evaluating this use-case in order to inform provision of public services for SARS-CoV-2 and potential future significant respiratory pandemics:

- Risks are very close to minimal at this stage in the pandemic
- The research is being conducted and was subject to the approval of Ministry of Health and Blantyre District health, recognising the impracticality and low participation of contact tracing during the early months of SARS-CoV-2 using means other than secondary distribution.
- This research component is designed to evaluate a public service program and could not practicably be carried out without the waiver or alteration given the nature of secondary distribution.
- Participants will be provided with information on self-testing and the project via information leaflets, including information on serious symptoms that require medical attention, and individual risk-factors that require special caution.
- A telephone number will be provided to allow consultation with a clinically qualified study team member who will also talk through all aspects relevant to a verbal consent process and stress voluntariness of participation.
- Potential harms will be further minimised by talking through the main risks and benefits and stressing the need for voluntary participation to the index patient during their written informed consent process.

Once antivirals are available in Malawi, information on eligibility criteria and how to access treatment will also be provided to index patients and their contacts.

Confidentially will be maintained by not recording any personal information (such as name or address) that could identify the contacts, with all information instead recorded by telephone interview that will just record at the *household level* how many adult contacts were eligible for testing, how many of those tested, how many of those had known clinical vulnerability (age over 65 years or age over 50 plus self-identified co-morbidity) and how many in each of these categories tested positive and what subsequent steps, if any, were taken to link to care and prevent onwards transmission (masks, isolation). Social harms and other adverse events will also be reported at household level (see below for category and severity).

We also request waiver of informed consent for use of routine programmatic data (extraction of testing registers) collected by the implementer team in compliance with routine COVID-19 testing and reporting standards for Malawi.

7. Anticipated benefits and harms

There are a number of potential benefits for participants, including the potential to receive early diagnosis for COVID-19 and masks to prevent onward transmission at home. The potential for harm is minimal, with COVID-19 testing scaled up globally, and self-testing considered Standard of Care in many high-income countries.

However, risks include potential for false positive and false negative results. These will be minimized by use of quality-assured kits stored in controlled conditions and by informing participants that negative tests do not exclude COVID-19 especially for patients with symptoms for longer than 7 days.

Social harms can arise from stigmatising reactions, especially for participants of the secondary distribution model of contact tracing who will be obliged to disclose their own positive status to contacts. These risks will be minimised by screening questions to identify patients experiencing ongoing intimate partner violence or who consider that they may be at risk of losing their job or told to leave their place of residence if their COVID-19 status was known. Participants will be prompted to consider their own risks before enrolling. High quality masks will be provided for contacts as well as participants. The process evaluation will help with identifying messages which mitigate risks and reduce potential harm.

Social harms will be considered and reported in the following categories: potential coercion to test, physical, economic, or emotional violence (resulting from a positive test), adverse mental health outcomes (resulting from a positive test or emotional violence), and physical pain from the test procedure including swab fracture resulting in retained foreign body, epistaxis, headache.

Within the secondary distribution model for self-test kits, we will actively screen for the potential of adverse events before we provide the test kits for distribution, and we ask about adverse events in our follow-up interview on day 8. There is also the unintended potential for the index patient to

screen children (under 18years old) as part of the secondary distribution of test kits. We will actively flag that these test kits are intended only for adult use in the initial index participant data tool, and we ask about use of kits at follow up.

Within the heath care worker screening use-case, participants testing positive will be expected to stay home. There could be a potential stigmatization from their colleagues or their employer, or, if the participant is expected to use their sick leave during isolation, they could potentially exhaust this sick leave, and lose income.

To mitigate this, we have negotiated with Blantyre District Health Office that participation will be voluntary for all employees, and refusal to participate will be reported and so not change the employee's relationship with the employer. No employee testing positive during this study will be unfairly treated (i.e., treated differently to any of their colleagues), or required to use up their sick leave while off work due to a positive self-test.

The written consent form for the healthcare worker study includes a discussion of risks to ensure that the informed consent requirements are met for employees consenting to be part of this process. Employer compliance will be assessed as part of the process evaluation.

We will also use lists of workers employed at each facility to provide a denominator of eligible participants for each clinic, but it is not required that every employee speaks to us. We will not seek information about paygrade or other sensitive matters, and will stress that participation is voluntary, but of particular benefit to individuals who may be eligible for antiviral treatment when this becomes available (age over 50 years with comorbidity) or for anyone who has a vulnerable household contact due to the benefit of early diagnosis and masking in reducing their risk of acquiring infection.

8. Conflict of interest

Neither the PI nor any of the collaborators has any conflict of interest.

9. Compensation for participants

Participants completing study activities lasting one hour will be compensated K3000 which includes transport to the venue of the activities.

10. Possible constraints

The main constraint that may directly impact the work described in this protocol relates to having no COVID-19 at all during the time of study implementation. This scenario is extremely unlikely given that the Malawi daily COVID-19 dashboard rarely reports zero cases. Moreover, we have made extremely modest assumptions regarding a) the number of positive COVID-19 cases to be detected in the non-randomized controlled study [25 in the standard of care and 35 in the intervention arm] b) the duration of any single wave – we assume every six months. Other less serious constraints relate to unacceptability of the study by the gate keepers including the district health office in Blantyre and other districts where we plan to conduct the project. We have already secured a letter of support from the Ministry of Health and have had positive meetings with District Health Management in all districts concerned culminating in letters of support.

11. Requirements

11.1. Personnel

A study coordinator will supervise the research / field team while the PI will provide overall leadership of the project. A health economist will lead the costing and cost effectiveness work assisted by a health economics assistant. Similarly, the process evaluation will be led by a senior social scientist with delegation of data collection to a social science assistant. The bulk of the data will be collected by field workers.

11.2. Materials and consumables

The following materials and consumables will be required for the study:

1. STANDARD Q COVID-19 Ag Test (SD Biosensor)
2. PanBio COVID-19 Ag Rapid Test Device (Abbott Rapid Diagnostics)
3. ACON Flow*flex*™ COVID-19 Antigen Home Test for self-testing
4. Writing materials, study materials, visual aids and clip boards will be required during protocol training and other training activities.
5. Printing
6. Vehicle running costs
7. KN95 masks
8. Surgical masks
9. Other PPE as appropriate

11.3. Equipment

Tablets for electronic data capture, lockable filing cabinets for temporary storage of completed consent forms and study tools.

11.4. Space

Space for storing study tools, equipment and consumables as well as private space for conducting study procedures will be required in all recruitment health facilities and other places where project components will be conducted.

11.5. Miscellaneous

Airtime for mobile communication between study personnel will be required.

12. Budgetary estimates

Table 6: Budget

No	Description							USD/MWK
1	**Personnel and training**	**Qty**	**Unit**		**Amount**	**Total (MWK)**	**USD**	**Comments**
	Research assistant	1	10 months	10	1,000,000.00	10,000,000.00	12,500.00	
	Field worker	5	4 months: surveys	3	320,000.00	4,800,000.00	6,000.00	
	Protocol training	2	Session	2	600,000.00	2,400,000.00	3,000.00	
	Translation and transcription	1	lampsum	1	100,000.00	100,000.00	125.00	
2	**Implementation costs**						-	
	Participant compensation	60	Qualitative: cognitive int	1	3,000.00	180,000.00	225.00	
	Participant compensation	2492	Feasibility survey	1	3,000.00	7,476,000.00	9,345.00	
	Vehicle hiring costs	1	4 months: lampsum	4	1,000,000.00	4,000,000.00	5,000.00	
	Mobile phone credit	1	4 months	4	100,000.00	400,000.00	500.00	
3	**Equipment & consumables**						-	
	Tablet	5	each	1	200,000.00	1,000,000.00	1,250.00	
	Office equipment	2	lampsum	1	300,000.00	600,000.00	750.00	
	PPE and COVID measures	1	lampsum	1	1,000,000.00	1,000,000.00	1,250.00	
	Digital recorders	3	each	1	100,000.00	300,000.00	375.00	
	COVID-19 Self-sampling kits	600	each - Donated	1	-	-	-	Donated
	COVID-19 RDT kits	6000	each - Donated	1	-	-	-	Donated
	COVID-19 Self-testing kits	2100	each - Donated	1	-	-	-	Donated
	PCR to confirm self-test +ve kits	50	each	1	40,000.00	2,000,000.00	2,500.00	
	Stationary	2	lampsum	1	500,000.00	1,000,000.00	1,250.00	
4	**Meetings**						-	
	Hospital engagement meetings	4	lampsum	1	150,000.00	600,000.00	750.00	
5	**Dissemination costs**						-	
	National dissemination	1	lampsum	1	225,000.00	225,000.00	281.25	
	MoH dissemination	1	lampsum	1	225,000.00	225,000.00	281.25	
6					**Total**	**36,306,000.00**	**45,382.50**	
7	**10% overheads in KUHeS**					**3,630,600.00**	**4,538.25**	
	**Overall total**					**39,936,600.00**	**49,920.75**	

13. Justification of budget

The main budget elements include personnel to run the project components including the overall leadership provided by the PI. Other important costs relate to procurement of test kits, training of implementation and research teams in vital aspects such as good clinical practice and protocol as well as participant compensation and meetings.

.

14. References

1. Ritchie H, Mathieu E, Rodés-Guirao L, Appel C, Giattino C, Ortiz-Ospina E, et al. Coronavirus Pandemic (COVID-19). Published online at OurWorldInData.org. 2020. [cited 2022 22 Sep 2022]. Available from: https://ourworldindata.org/coronavirus.
2. Estimating excess mortality due to the COVID-19 pandemic: a systematic analysis of COVID-19-related mortality, 2020-21. Lancet (London, England). 2022;399(10334):1513-36.
3. World Health Organization (WHO). The true death toll of COVID-19: estimating global excess mortality Geneva, Switzerland: World Health Organization; 2022 [updated 9 March 2022.
4. Estimating global, regional, and national daily and cumulative infections with SARS-CoV-2 through Nov 14, 2021: a statistical analysis. Lancet (London, England). 2022;399(10344):2351-80.
5. World Health Organization (WHO). Therapeutics and COVID-19: living guideline. Geneva, Switzerland: World Health Organization; 2022 16 Sep 2022.
6. Madhi SA, Kwatra G, Myers JE, Jassat W, Dhar N, Mukendi CK, et al. Population Immunity and Covid-19 Severity with Omicron Variant in South Africa. The New England journal of medicine. 2022;386(14):1314-26.
7. Drain PK. Rapid Diagnostic Testing for SARS-CoV-2. The New England journal of medicine. 2022;386(3):264-72.
8. Dinnes J, Deeks JJ, Berhane S, Taylor M, Adriano A, Davenport C, et al. Rapid, point-of-care antigen and molecular-based tests for diagnosis of SARS-CoV-2 infection. The Cochrane database of systematic reviews. 2021;3(3):Cd013705.
9. Nkengasong J. Let Africa into the market for COVID-19 diagnostics. Nature. 2020;580(7805):565.
10. Mina MJ, Andersen KG. COVID-19 testing: One size does not fit all. Science. 2021;371(6525):126-7.
11. Singanayagam A, Patel M, Charlett A, Lopez Bernal J, Saliba V, Ellis J, et al. Duration of infectiousness and correlation with RT-PCR cycle threshold values in cases of COVID-19, England, January to May 2020. Euro surveillance : bulletin Europeen sur les maladies transmissibles = European communicable disease bulletin. 2020;25(32).
12. World Health Organization. Use of SARS-CoV-2 antigen-detection rapid diagnostic tests for COVID-19 self-testing. Interim guidance. Geneva, Switzerland: WHO; 2022.
13. Indravudh PP, Choko AT, Corbett EL. Scaling up HIV self-testing in sub-Saharan Africa: a review of technology, policy and evidence. Curr Opin Infect Dis. 2018;31(1):14-24.
14. Choko AT, Desmond N, Webb EL, Chavula K, Napierala-Mavedzenge S, Gaydos CA, et al. The uptake and accuracy of oral kits for HIV self-testing in high HIV prevalence setting: a cross-sectional feasibility study in Blantyre, Malawi. PLoS medicine. 2011;8(10):e1001102.
15. Johnson CC, Kennedy C, Fonner V, Siegfried N, Figueroa C, Dalal S, et al. Examining the effects of HIV self-testing compared to standard HIV testing services: a systematic review and meta-analysis. Journal of the International AIDS Society. 2017;20(1):21594.
16. Stohr J, Zwart VF, Goderski G, Meijer A, Nagel-Imming CRS, Kluytmans-van den Bergh MFQ, et al. Self-testing for the detection of SARS-CoV-2 infection with rapid antigen tests for people with suspected COVID-19 in the community. Clinical microbiology and infection : the official publication of the European Society of Clinical Microbiology and Infectious Diseases. 2021.
17. Lindner AK, Nikolai O, Rohardt C, Kausch F, Wintel M, Gertler M, et al. Diagnostic accuracy and feasibility of patient self-testing with a SARS-CoV-2 antigen-detecting rapid test. Journal of clinical virology : the official publication of the Pan American Society for Clinical Virology. 2021;141:104874.
18. NHS; UK. Your step-by-step guide to DHSC COVID-19 Self-Test (Rapid Antigen Test). NHS Test and Trace: NHS; 2022.
19. Simwinga M, Kumwenda MK, Dacombe RJ, Kayira L, Muzumara A, Johnson CC, et al. Ability to understand and correctly follow HIV self-test kit instructions for use: applying the cognitive

interview technique in Malawi and Zambia. Journal of the International AIDS Society. 2019;22 Suppl 1(Suppl Suppl 1):e25253.
20. Woloshin S, Dewitt B, Krishnamurti T, Fischhoff B. Assessing How Consumers Interpret and Act on Results From At-Home COVID-19 Self-test Kits: A Randomized Clinical Trial. JAMA internal medicine. 2022.
21. Hammond J, Leister-Tebbe H, Gardner A, Abreu P, Bao W, Wisemandle W, et al. Oral Nirmatrelvir for High-Risk, Nonhospitalized Adults with Covid-19. The New England journal of medicine. 2022;386(15):1397-408.
22. Wood BR, Ballenger C, Stekler JD. Arguments for and against HIV self-testing. Hiv/Aids. 2014;6:117-26.
23. Sande LA, Matsimela K, Mwenge L, Mangenah C, Choko AT, d'Elbée M, et al. Costs of integrating HIV self-testing in public health facilities in Malawi, South Africa, Zambia and Zimbabwe. BMJ Global Health. 2021;6(Suppl 4):e005191.
24. Wachinger J, Olaru ID, Horner S, Schnitzler P, Heeg K, Denkinger CM. The potential of SARS-CoV-2 antigen-detection tests in the screening of asymptomatic persons. Clinical microbiology and infection : the official publication of the European Society of Clinical Microbiology and Infectious Diseases. 2021;27(11):1700.e1-.e3.
25. Albert E, Torres I, Bueno F, Huntley D, Molla E, Fernandez-Fuentes MA, et al. Field evaluation of a rapid antigen test (Panbio COVID-19 Ag Rapid Test Device) for COVID-19 diagnosis in primary healthcare centres. Clin Microbiol Infect. 2020.
26. Chaimayo C, Kaewnaphan B, Tanlieng N, Athipanyasilp N, Sirijatuphat R, Chayakulkeeree M, et al. Rapid SARS-CoV-2 antigen detection assay in comparison with real-time RT-PCR assay for laboratory diagnosis of COVID-19 in Thailand. Virology journal. 2020;17(1):177.
27. van Beek J, Igloi Z, Boelsums T, Fanoy E, Gotz H, Molenkamp R, et al. From more testing to smart testing: data-guided SARS-CoV-2 testing choices. Preprint https://wwwmedrxivorg/content/101101/2020101320211524v2. 2021 Preprint.
28. World Health Organization (WHO). COVID-19 Target product profiles for priority diagnostics to support response to the COVID-19 pandemic v.1.0 Geneva, Switzerland: World Health Organization; 2020 [updated 28 Sep 2020; cited 2022 1 June 2022]. Available from: https://www.who.int/publications/m/item/covid-19-target-product-profiles-for-priority-diagnostics-to-support-response-to-the-covid-19-pandemic-v.0.1.
29. Berger A, Nsoga MTN, Perez-Rodriguez FJ, Aad YA, Sattonnet-Roche P, Gayet-Ageron A, et al. Diagnostic accuracy of two commercial SARS-CoV-2 antigen-detecting rapid tests at the point of care in community-based testing centers. PloS one. 2021;16(3):e0248921.
30. Foundation for Innovative New Diagnostics (FIND). FIND Evaluation of Acon Biotech (Hangzhou) Co. Ltd. Flowflex SARS-CoV-2 Antigen Rapid Test. External Report. Version 1.0 Geneva, Switzerland: FIND; 2021 [updated June 2021; cited 2022 1 June 2022]. Available from: https://www.finddx.org/wp-content/uploads/2021/06/Acon_Flowflex_Ag-Public-Report_v1_20200609.pdf.
31. Malawi Ministry of Health. Recommended testing algorithm for COVID-19 antigen rapid diagnostic test for Malawi - November 2022. Lilongwe, Malawi: Malawi Ministry of Health; 2020 2020.
32. Malawi Ministry of Health. Guidance on home-based management of persons with asymptomatic and mild COVID-19. Lilongwe, Malawi: Malawi Ministry of Health; 2020 2020.
33. World Health Organization. Contact tracing in the context of COVID-19 Geneva, Switzerland: WHO; 2021 [Available from: https://www.who.int/publications/i/item/contact-tracing-in-the-context-of-covid-19.
34. Malawi Government, Helath. Mo. COVID-19 Case management manual (3rd Edition). 2021.
35. R Core team. R: A language and environment for statistical computing. R Foundation for Statistical Computing, Vienna, Austria. URL http://www.R-project.org/. 2015.

15. List of Appendices

Appendix 1A – NC06A Participant Information Leaflet, Informed Consent Form for Contact Tracing

Appendix 1B – NC06B Information Flyer on 3ACP research project

Appendix 2 – NC07 Participant Information Leaflet, Informed Consent Form for Healthcare Workers

Appendix 3A – NC08A Participant Information Leaflet, Informed Consent Form for Performance Evaluation (OPD setting)

Appendix 3B – NC08B Participant Information Leaflet, Informed Consent Form for Performance Evaluation (congregate setting)

Appendix 3D – NC08D Participant Information Leaflet, Informed Consent Form for Performance Evaluation (Implementers)

Appendix 4 – NC09 Information sheet & consent form for Client Cost Exit Survey

Appendix 5 – NC10 Information sheet & consent form: HCW Cost time-motion survey

Appendix 6 – NC03 participant information sheet and informed consent form:

Key Informant Interview

Appendix 7 – CH00: HCW Eligibility

Appendix 8 – CH01A: HCW Baseline Questionnaire

Appendix 9 – CH02: HCW Follow-up Questionnaire

Appendix 10 – CH03: HCW Exit Questionnaire

Appendix 11 – CP00 OPD Attendees' Questionnaire

Appendix 12 – CP01 OPD attendees Questionnaire

Appendix 13 – CW00 Congregate Setting Eligibility Screening Form

Appendix 14 – CW01 Congregate Setting Questionnaire

Appendix 15 – CC00 Index Eligibility

Appendix 16 – CC01 Index client baseline questionnaire

Appendix 17 – CC02 Index Follow-up questionnaire

Appendix 18 – Clients Semi structured Interviews topic guide

Appendix 19 – CK01 Key Informant Interviews topic guide

Appendix 20 – Providers Semi structured Interviews topic guide

Appendix 22 – Facility Costing Data collection tool

Appendix 23 – Client Costs Exit Questionnaire
