# Supplementary material for: COVID-19 diagnosis within five days of symptoms onset among healthcare workers in Malawi; Non-randomized control trial of self-testing using Ag-RDTs
Source: PLOS Glob Public Health. 2025 Dec 16;5(12):e0005604. doi: 10.1371/journal.pgph.0005604 (PMC12707653; doi:10.1371/journal.pgph.0005604)
Supplement: S1 Checklist — (DOC) [file pgph.0005604.s005.doc]

# Inclusivity in global research

PLOS’ policy on inclusivity in global research aims to improve transparency in the reporting of research performed outside of researchers’ own country or community and ensures that PLOS publications reporting global research adhere to high standards for research ethics and authorship. Authors of relevant research articles may be asked to complete the questionnaire below, which outlines ethical, cultural, and scientific considerations specific to inclusivity in global research. This questionnaire may be requested when researchers have travelled to a different country to conduct research, if research uses samples collected in another country, research with Indigenous populations or their lands, or if research is on cultural artefacts. Researchers travelling to another country solely to use laboratory equipment will not normally be required to complete the questionnaire. However, the questionnaire can be requested at the journal’s discretion for any submission – if you have been requested to complete this questionnaire by the PLOS journal you submitted to, please do so.

Please complete the questionnaire below and include this as a Supporting Information file with your manuscript. Note that if your paper is accepted for publication, this checklist will be published with your article in the supporting information files. Please ensure that you reference the checklist in the main body of your manuscript. We suggest adding a subsection ‘Inclusivity in global research’ to your Methods section and adding the following sentence: “Additional information regarding the ethical, cultural, and scientific considerations specific to inclusivity in global research is included in the Supporting Information (SX Checklist)”

The questions have been designed to be applicable to a wide range of study types, and there are subsections for both human subjects research and non-human subjects research. If any of the questions are not relevant to your research please mark them as “N/A” as appropriate.

**Ethical considerations, permits and authorship**

*This section is applicable to all research types.*

Provide details as to who granted permissions and/or consent for the study to take place in the Methods section of your manuscript. This should include the names of **all** ethics boards, governmental organizations, community leaders or other bodies that provided approval for the study. If individuals provided approval refer to these people by their role or title but do not list their name(s).

Reported on page number: 11- *Ethics approval for the study was obtained from Kamuzu University of Health Science’s College of Medicine Research and Ethics Committee (COMREC) in Malawi (P.05/22/3649), and internationally from the World Health Organization Ethics Review Committee in Geneva, Switzerland (CERC.0163), and the London School of Hygiene & Tropical Medicine Ethics Committee (26874). Permission to access the 12 participating health facilities was obtained from the director of health and social services for Blantyre.*

If there were any deviations from the study protocol after approval was obtained please provide details of these changes in the Methods section of your manuscript.

Reported on page number: N/A *(No protocol deviation recorded during the study implementation)*

Did this study involve local collaborators that are residents of the country where the research was conducted or members of the community studied? If you do not have any authors from said communities, please provide an explanation for this below.

*The study involved several local collaborators from Malawi comprising of implementers from Family Health Services (FHS) in Malawi, Blantyre and researchers from Malawi Liverpool Wellcome programme and Kamuzu University of Health Sciences . All authors from these institutions are listed among the list of authors in the manuscript*

Everyone listed as an author should meet PLOS’ criteria for authorship and all individuals who meet these criteria should be included in the author byline, rather than the acknowledgements. For further information please see the journal’s Authorship Policy.

**Human subjects research (e.g. health research, medical research, cross-cultural psychology)**

Did you obtain written informed consent from a representative of the local community or region before the research took place? How did you establish who speaks for the community? Details of written informed consent obtained from study participants should be reported separately in the Methods section of your manuscript.

*No, written informed consent was not obtained from a community representative prior to the research. However, written permission to conduct the study was obtained from the director of health and social services. Further more, the study was presented to the community scientific advisory board for Malawi Liverpool Wellcome program which comprise of different community representatives from within Blantyre.*

How did members of the local community provide input on the aims of the research investigation, its methodology, and its anticipated outcome(s)?

*Yes. Firstly, before the study protocol was finalized, the study was presented to the Blantyre Ditrict Health Office research committee, which recommended the 12 health facilties to participate in the study based on need for COVID-19 testig. The study protocol was later presented to the ministry of health diagnostics technical working group, who suggested routine quality control of test kits and led in the diagnostic accuracy of the test kits before implementation. Furthermore, implemetation of the project required development of COVID-19 prevention messages which were not in existence at the time the study was being conducted. This activity was led by our implementation particners from Family Heath Services, the human centered design approach was adopted to get insights on barriers and motivators surround the uptake of COVID-19 testing from healthcare workers, community members and other key stakeholders.*

When engaging with the local community, how did you ensure that the informed consent documents and other materials could be understood by local stakeholders?

*All study information sheets and consent forms were translated into Chichewa, the main local language in Malawi. Trained research assistants fluent in both English and Chichewa, obtained the consent and explain the study to the potential participant verbally, providing all pertinent information (purpose, procedures, risks, benefits, alternatives to participation, etc.) using the appropriate information sheet for the respective use case. Potential participants were given ample opportunity to ask questions and had all of their questions answered. Each participant was provided with a written information sheet to read, if they could not read, the research assistant read out the consent for them. Potential participans were offered time to consider if they want to participate in the study and were assessed for comprehension of the study by asking them to describe the study purpose and what is expected from participants.*

Will the findings of the research be made available in an understandable format to stakeholders in the community where the study was conducted (e.g. via a presentation, summary report, copies of publications, etc.)? Please provide details of how this will be achieved.

*Yes, dissemination meetings were held at each of the participating health facility, to brief all stakeholders including government agencies, governing bodies of patent medicine vendors, pharmacist, representatives of study areas and communities, local researchers, local media, representative of study sites of the study findings and the implication for policy change in Nigeria and globally.*

**Non-human subjects research using specimens/ animals collected as part of the study, or those housed in archival collections. Examples include archaeology, paleontology, botany and zoology.**

Did the permission you obtained from a local authority to perform the study include an agreement on access to outputs and benefit sharing? This may include procedures to enable fair distribution of the benefits and resources arising from the research performed. Please include any details of Prior Informed Consent and Benefit Sharing Agreements obtained. These may be required by field-specific regulations, for example the Convention on Biological Diversity (CBD) and the associated Nagoya Protocol.

*No spacemens were archived from the study. All materials used for testrning were discrded after the test.*

If the material used in your study was imported, please A) provide the year it was imported and B) indicate whether permits were obtained to import/export the materials used, C) provide details of any permits obtained. If this information is not available, please indicate this.

*The rapid diagnostic test kits were imported in December 2022. In compliance with Malawi’s regulatory guidelines for the importation of in vitro diagnostic devices, an import waiver/custom clearance was obtained from the Malawi Revenue Authority (MRA) and all logistics were handled by FHS, the implementation partiners*

If you used archival specimens, please state how the material used in your study was acquired by the institute it is held in and provide details of any permits obtained for the original excavations/ sample collection. If this information is not available, please indicate this.

*No, archive specimen were not used for this study*

How was the potential cultural significance of the materials collected in your study to local communities considered in your research design? Were Indigenous peoples and/or local researchers and institutions involved with archaeological excavations / collection of specimens? If so, please provide a description of their involvement.

*The study did not involve the collection of culturally significant materials, archaeological excavations, or biological specimens. The study used nasal swabs collected from people attending the outpatient departments and healthcare workers doing self-testing*

If your manuscript includes photographs of human remains please indicate whether authors obtained permission from descendants or affiliated cultural communities to do so.

*N/A*
